# Meta-analyses of the effects of high-intensity interval training in elite athletes—part I: mean effects on various performance measures

**DOI:** 10.3389/fphys.2024.1486526

**Published:** 2025-01-03

**Authors:** Hans-Peter Wiesinger, Thomas Leonard Stöggl, Nils Haller, Julia Blumkaitis, Tilmann Strepp, Francesca Kilzer, Anna Schmuttermair, Will G. Hopkins

**Affiliations:** ^1^ Department of Sport and Exercise Science, Paris Lodron University Salzburg, Salzburg, Austria; ^2^ Institute of Nursing Science and Practice, Center for Public Health and Healthcare Research, Paracelsus Medical University, Salzburg, Austria; ^3^ Institute of General Practice, Family Medicine and Preventive Medicine, Center for Public Health and Healthcare Research, Paracelsus Medical University, Salzburg, Austria; ^4^ Red Bull Athlete Performance Center, Thalgau, Austria; ^5^ Department of Sports Medicine, Rehabilitation and Disease Prevention, Johannes Gutenberg University, Mainz, Germany; ^6^ Internet Society for Sport Science, Auckland, New Zealand

**Keywords:** meta-regression, endurance, sprint, performance, interval training, elite athletes

## Abstract

**Introduction:**

Meta-analysts have found that high-intensity interval training (HIIT) improves physical performance, but limited evidence exists regarding its effects on highly trained athletes, measures beyond maximum oxygen uptake ( $\dot{V}$ O_2max_), and the moderating effects of different types of HIIT. In this study, we present meta-analyses of the effects of HIIT focusing on these deficits.

**Methods:**

The effects of 6 types of HIIT and other moderators were derived from 34 studies involving highly trained endurance and elite athletes in percent units via log-transformation from separate meta-regression mixed models for sprint, time–trial, aerobic/anaerobic threshold, peak speed/power, repeated-sprint ability, $\dot{V}$ O_2max_, and exercise economy. The level of evidence for effect magnitudes was evaluated based on the effect uncertainty and the smallest important change of 1%.

**Results:**

Compared with control training, HIIT showed good to excellent evidence for the substantial enhancement of most measures for some athlete subgroups in practically important study settings defined by effect moderators (maximum of 12.6%, for endurance female athletes after 6 weeks of aerobic traditional long intervals). The assessment of the moderators indicated good evidence of greater effects as follows: with more aerobic types of HIIT for $\dot{V}$ O_2max_ (+2.6%); with HIIT added to conventional training for most measures (+1.1–2.3%); during the competition phase for $\dot{V}$ O_2max_ (+4.3%); and with tests of longer duration for sprint (+5.5%) and time trial (+4.9%). The effects of sex and type of athlete were unclear moderators. The heterogeneity of HIIT effects within a given type of setting varied from small to moderate (standard deviations of 1.1%–2.3%) and reduced the evidence of benefit in some settings.

**Conclusion:**

Although athletes in some settings can be confident of the beneficial effects of HIIT on some measures related to competition performance, further research is needed. There is uncertainty regarding the mean effects on exercise economy and the modifying effects of sex, duration of intervention, phase of training, and type of HIIT for most measures.

**Systematic Review Registration:**

https://www.crd.york.ac.uk/PROSPERO/display_record.php?RecordID=236384.

## 1 Introduction

High-intensity interval training (HIIT) has been studied extensively for its effects on endurance performance and the physiology of healthy adult individuals. There have been sufficient studies to warrant six meta-analyses showing substantial effects on $\dot{V}$ O_2max_ (Gist et al., 2014; Sloth et al., 2013; Bacon et al., 2013; Weston et al., 2014; Milanovic et al., 2015), mean power in time trials (Carr et al., 2011), or peak power in 30-s Wingate tests (Weston et al., 2014). While most of these meta-analyses have not explored subgroup analysis or other moderator effects (Gist et al., 2014; Sloth et al., 2013; Bacon et al., 2013; Carr et al., 2011), two have indicated that the more trained an individual, the smaller the improvement in $\dot{V}$ O_2max_ (Weston et al., 2014; Milanovic et al., 2015), and in one of these studies, there was a substantial but unclear effect of HIIT on low-level athletes (Weston et al., 2014). No previous meta-analysis has focused on the effect of HIIT on elite athletes, who may be training close to an optimum level and, therefore, might gain less extra benefit from HIIT. Furthermore, HIIT encompasses different types that impose varying degrees of stress on the different systems mediating performance (Billat, 2001), but previous meta-analyses have focused primarily on $\dot{V}$ O_2max_ without investigating the modifying effects of different types of HIIT on this and other performance measures.

**FIGURE 1:**
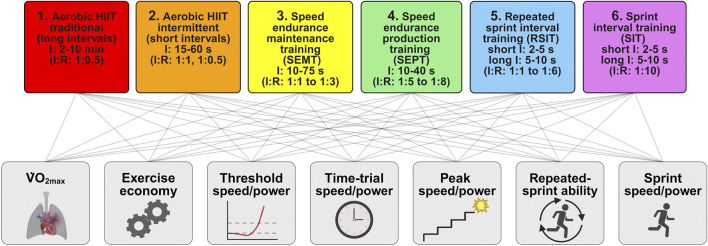
Schema for the classification of types of high-intensity interval training and their presumed effects on the measures and predictors of performance. The types of HIIT (upper row) and performance (lower row) are ordered from most aerobic on the left to most anaerobic on the right. Connecting lines of these rows show the possibility that all types of HIIT affect all the measures and predictors of endurance performance. I, interval duration; I:R, interval to recovery ratio; $\dot{V}$ O_2max_, maximum oxygen uptake (created using BioRender.com).

To evaluate the effects of different types of HIIT, we categorized HIIT into six different types [Figure 1, based on the toolbox in the study by Stöggl et al. (2024)], ranging from most aerobic to most anaerobic. Aerobic intervals are performed with submaximal effort, while anaerobic intervals are performed at maximum effort and last up to 75 s (Gastin, 2001). Aerobic intervals are further categorized into traditional long intervals with durations of 2–10 min (Seiler et al., 2013; Spencer et al., 1996; Sandbakk et al., 2013) and intermittent short intervals with durations of 15–60 s (Helgerud et al., 2007; Stöggl et al., 2023). For anaerobic intervals, we included an intermediate level: speed endurance training (SET) and sprint interval training (SIT). For SET, intervals last between 10 and 75 s (Iaia and Bangsbo, 2010; Mohr et al., 2007); for SIT, sprints last between 2 and 10 s to ensure reaching maximal acceleration, speed, or power (Girard et al., 2011; Iaia et al., 2009; Bishop et al., 2011). SET can be differentiated into two types of HIIT: speed endurance maintenance training (SEMT) and speed endurance production training (SEPT), with the latter having ∼2 times longer recovery periods than the former (Iaia and Bangsbo, 2010). Sprint intervals can be divided into repeated sprint interval training (RSIT) and sprint interval training (SIT), again differentiated by the duration of recovery periods (Iaia and Bangsbo, 2010).

In their quest to optimize the training of competitive athletes, researchers have investigated the effects of several types of HIIT on various measures of performance: sprints and repeated sprints for team-sport athletes and time trials for athletes competing as individuals in a range of sports. Predictors of endurance performance have also been investigated: $\dot{V}$ O_2max_, peak speed or power in an incremental test, time to exhaustion in a constant-power or constant-speed test, aerobic/anaerobic threshold speed or power, and exercise economy. There have been only two studies of the effect of HIIT on time to exhaustion on elite athletes (Smith et al., 2003; Breil et al., 2010); in any case, effects on time to exhaustion should be converted to effects on power output to permit practical application to competitive athletes, and the conversion is problematic (Hinckson and Hopkins, 2005). However, there have been enough studies to permit the meta-analysis of the effects of HIIT on each of the other measures and predictors of performance. These measures and predictors are shown in Figure 1, ordered approximately to reflect the presumed greater effects of aerobic HIIT on aerobic measures and greater effects of anaerobic HIIT on anaerobic measures (to the left and right of the figure, respectively). The possibility that all types of HIIT affect all the measures is shown by the lines connecting HIIT with the measures. Our meta-analyses represent pioneering work in HIIT research in three respects: they are the first meta-analyses focusing on highly trained elite athletes; they include the modifying effects of different types of HIIT; and the effects on performance have been assessed with seven measures linked in varying degrees with aerobic power, anaerobic power, the neuromuscular system, and efficiency.

## 2 Methods

### 2.1 Study registration and eligibility criteria

We pre-registered our meta-analysis at PROSPERO (ID number: CRD42021236384) and followed the PRISMA guidelines (Page et al., 2021). In the analysis, separate effects were estimated for HIIT and for conventional training as control, then combined to estimate the net effect of HIIT; we, therefore, also included studies of HIIT without control groups because the controlled trials effectively provided controls for these studies (Weston et al., 2014). Studies were eligible for inclusion if the outcome was some measure of endurance or sprint performance in highly trained endurance athletes [runners (Smith et al., 2003; Stöggl and Sperlich, 2014; Menz et al., 2015; Salazar-Martinez et al., 2018), cyclists (Stöggl and Sperlich, 2014; Menz et al., 2015; Salazar-Martinez et al., 2018; Clark et al., 2014; Hanstock et al., 2020; Laursen et al., 2002; Skovereng et al., 2018; Stenqvist et al., 2020; Sylta et al., 2016; Rønnestad and Vikmoen, 2019; Stepto et al., 1999), duathletes or triathletes (Smith et al., 2003; Stöggl and Sperlich, 2014; Menz et al., 2015; Salazar-Martinez et al., 2018; Hanstock et al., 2020; Laursen and Jenkins, 2002), cross-country skiers (Sandbakk et al., 2013; Stöggl and Sperlich, 2014; Johansen et al., 2021; Sandbakk et al., 2011), and rowers (Stevens et al., 2015)], with mean baseline $\dot{V}$ O_2max_ values ≥60 mL kg^–1^·min^–1^ for men (Sandbakk et al., 2013; Smith et al., 2003; Stöggl and Sperlich, 2014; Menz et al., 2015; Salazar-Martinez et al., 2018; Clark et al., 2014; Hanstock et al., 2020; Laursen et al., 2002; Skovereng et al., 2018; Stenqvist et al., 2020; Sylta et al., 2016; Rønnestad and Vikmoen, 2019; Stepto et al., 1999; Johansen et al., 2021; Sandbakk et al., 2011; Stevens et al., 2015) and ≥55 mL kg^–1^·min^–1^ for women (Sandbakk et al., 2013; Stöggl and Sperlich, 2014; Menz et al., 2015; Sandbakk et al., 2011), or in healthy other elite athletes [first league, national team, or international level in ball (Wells et al., 2014; Helgerud et al., 2001; Akdoğan et al., 2021; Iaia et al., 2015; Purkhus et al., 2016; Soares-Caldeira et al., 2014; Thomassen et al., 2010; Venturelli et al., 2008; Chtara et al., 2017; Hermassi et al., 2018; Selmi et al., 2018; Delextrat et al., 2018; Dupont et al., 2004), racket (Liu et al., 2021), canoe sports (Sheykhlouvand et al., 2018a; Sheykhlouvand et al., 2018b; Sheykhlouvand et al., 2016; Yang et al., 2017), and alpine skiers (Breil et al., 2010)]. We included one study on the upper-body exercise of male endurance athletes with $\dot{V}$ O_2max_ values of ∼55 mL kg^–1^·min^–1^ as their running $\dot{V}$ O_2max_ values met our inclusion criteria (Johansen et al., 2021). We accepted endurance athletes from different disciplines but did not include a mixture of endurance and other types of athletes within a single study. Studies in which training and testing were unrelated to the specific sport under investigation were excluded (Seo et al., 2019; Ojeda-Aravena et al., 2021; Herrera-Valenzuela et al., 2021; Thom et al., 2020; Sarkar et al., 2021). There was no eligibility for age, sports, training periodization, sample size, or international ranking of a national sports team. For two research groups that each presented the same outcomes for the same training and athletes in separate studies, the more recent study was excluded (Stöggl and Bjorklund, 2017; Laursen et al., 2005). Two different training studies of partially the same athletes were included as separate studies (Menz et al., 2015; Salazar-Martinez et al., 2018). The inclusion criteria for the HIIT interventions were at least two training sessions per week for at least a week and at least five sessions. Measures of $\dot{V}$ O_2max_ estimated from time trials were excluded. HIIT studies that included other types of interventions (e.g., ingestion of stimulants or other supplements, change in the environmental condition, or the use of modalities like compression garments) were excluded. HIIT exercise intensities had to be ≥90% of the maximal heart rate (Sandbakk et al., 2013; Breil et al., 2010; Stöggl and Sperlich, 2014; Menz et al., 2015; Johansen et al., 2021; Sandbakk et al., 2011; Helgerud et al., 2001; Seo et al., 2019), ≥90% of $\dot{V}$ O_2max_ (Smith et al., 2003; Salazar-Martinez et al., 2018; Sheykhlouvand et al., 2018b; Sheykhlouvand et al., 2016; Yang et al., 2017), or classified as “all-out” efforts for speed (Clark et al., 2014; Hanstock et al., 2020; Wells et al., 2014; Akdoğan et al., 2021; Iaia et al., 2015; Purkhus et al., 2016; Soares-Caldeira et al., 2014; Thomassen et al., 2010; Venturelli et al., 2008; Chtara et al., 2017; Hermassi et al., 2018; Selmi et al., 2018; Delextrat et al., 2018; Dupont et al., 2004) or power (Laursen et al., 2002; Skovereng et al., 2018; Stenqvist et al., 2020; Sylta et al., 2016; Rønnestad and Vikmoen, 2019; Stepto et al., 1999; Stevens et al., 2015; Sheykhlouvand et al., 2018a). The outcomes for several groups in multi-arm studies were excluded due to low exercise intensity (Supplementary Tables S3–S9). We accepted peer-reviewed articles published in English or German.

### 2.2 Literature search and data synthesis

**FIGURE 2:**
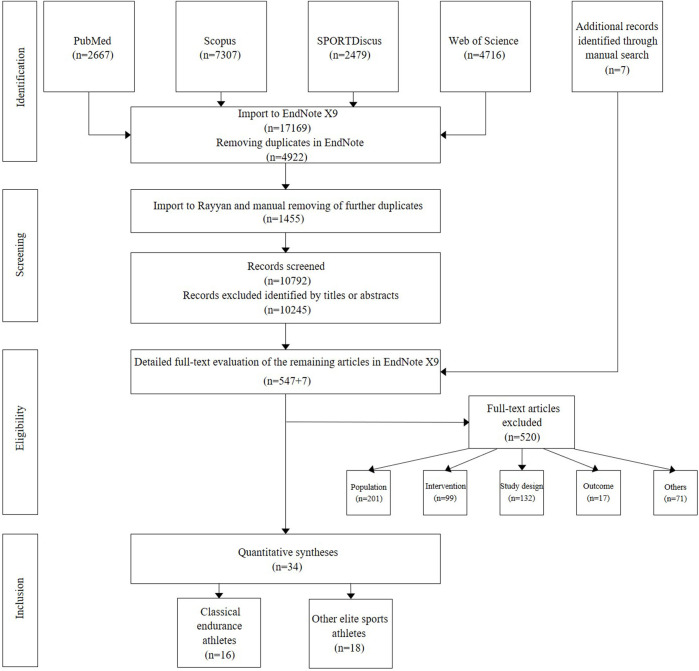
PRISMA flow diagram detailing the search included and excluded studies. A total of 34 files fulfilled the eligibility criteria (see references in Supplementary Appendix).

The workflow is presented in Figure 2. On 18 February 2021, the electronic bibliographic databases PubMed, Scopus, SPORTDiscus, and Web of Science were scanned for relevant studies. The search string, conducted in the title, abstract, and keywords, is presented in Supplementary Appendix. The search results were imported into EndNote 20.1 (Clarivate Analytics, Philadelphia, PA, United States) for deduplication before being exported to Rayyan QCRI software (https://rayyan.qcri.org). After another deduplication, all authors independently screened the articles based on the title and abstract, and we identified 547 papers that met the inclusion criteria. Seven additional studies identified through references cited in eligible articles were checked for inclusion. Subsequently, these full-text articles were screened, and discrepancies in 28 studies were resolved by two authors (HPW and JB).

### 2.3 Study quality

Two authors (HPW and JB) independently rated the included studies on a quality scale proposed by Galna et al. (2009) (Supplementary Tables S1, S2). Interrater discrepancies were resolved by consensus, and no studies were excluded due to low scores.

### 2.4 Data extraction

Three authors (JB, TS, and HPW) collected subject characteristics and quantitative data from pre- and post-tests. Two authors (WGH and HPW) searched for inferential statistics to obtain standard errors of each study estimate, decided on potential moderators, and conducted all statistical analyses and data presentations using Statistical Analysis System (SAS OnDemand for Academics, version 9.4, SAS Institute, Cary, NC). Raw data, when available (Stöggl and Sperlich, 2014; Clark et al., 2014) or digitizable (Breil et al., 2010; Skovereng et al., 2018; Rønnestad and Vikmoen, 2019; Sandbakk et al., 2011; Stevens et al., 2015; Purkhus et al., 2016; Thomassen et al., 2010) (DigitizeIt, 38108 Braunschweig, Germany, or WebPlotDigitizer Pacifica, CA, United States), were re-analyzed using the statistical model in the study using SPSS Statistics V.27.0 (IBM Corporation, Chicago, Illinois, United States). Some study estimates and uncertainties were digitized from figures (Sandbakk et al., 2013; Smith et al., 2003; Hanstock et al., 2020; Laursen et al., 2002; Stepto et al., 1999; Sandbakk et al., 2011; Iaia et al., 2015; Liu et al., 2021; Sheykhlouvand et al., 2016; Yang et al., 2017); the accuracy of data extraction was verified by re-digitization (HPW) and conspicuousness checks (WGH and HPW). Otherwise, HPW asked corresponding and/or co-authors for the required values (Smith et al., 2003; Menz et al., 2015; Salazar-Martinez et al., 2018; Hanstock et al., 2020; Stenqvist et al., 2020; Rønnestad and Vikmoen, 2019; Sandbakk et al., 2011; Stevens et al., 2015; Helgerud et al., 2001; Akdoğan et al., 2021; Chtara et al., 2017; Hermassi et al., 2018; Delextrat et al., 2018; Liu et al., 2021; Seo et al., 2019). Sufficient data for some variables were obtained (Menz et al., 2015; Salazar-Martinez et al., 2018; Hanstock et al., 2020; Skovereng et al., 2018; Stenqvist et al., 2020; Rønnestad and Vikmoen, 2019; Akdoğan et al., 2021; Liu et al., 2021), but responses were not received from some authors (Smith et al., 2003; Stevens et al., 2015; Helgerud et al., 2001; Chtara et al., 2017; Hermassi et al., 2018; Delextrat et al., 2018), while other authors had either deleted their data due to national regulations (Sandbakk et al., 2013; Sandbakk et al., 2011) or could not send the required information for all requests (Akdoğan et al., 2021; Liu et al., 2021).

Endurance and sprint performance measures were categorized into sprint speed/power, repeated-sprint ability, time–trial speed/power, peak speed/power, aerobic/anaerobic threshold, $\dot{V}$ O_2max_, and exercise economy (Figure 1). Due to a scarcity of intervention studies on different types of athletes (classical endurance or other elite athletes and male or female participants), meta-analyses for some outcome measures were restricted to specific subgroups. For sprint speed/power, the meta-analysis was limited to non-endurance athletes and based on the mean speed of linear running between 10 m and 40 m. The longest sprint duration was 7.69 s for a 40-m sprint (Selmi et al., 2018). For the repeated-sprint-ability tests, the distances were between 20 m and 40 m, sometimes including a 180° turn halfway. The number of sprints was between 2 and 15, with a mean time of a single sprint of 6.0 ± 1.4 s and a total sprint time of 44 ± 17 s. The mean or total time was used for the percent effects of the interventions. Time-trial times were all for endurance athletes and lasted between 0.5 min and ∼1 h. The trials consisted of either fixed-distance (0.4 km–40 km) or fixed-time (40 min) running or cycling. Wingate tests on a cycle or rowing ergometer of 30 s or 60 s were also categorized as time trials.

Performance effects of the time trial were obtained via changes in the mean speed or power. For this, we converted the percent effects on cycling and rowing time or speed to percent effects on power by multiplying by 2.2 for cycling and 3.0 for rowing (Hopkins et al., 2001). For peak speed/power, we used the highest speed obtained during running incremental treadmill ramp tests or YoYo Intermittent Recovery running tests at Level 1 or 2 (YoYo IR1 and IR2) or the highest power during an incremental ramp ergometer cycle test. The aerobic and anaerobic threshold data were presented by the authors of the included studies as speed or power. When fractional utilization at the threshold was presented (Sandbakk et al., 2013; Smith et al., 2003; Sandbakk et al., 2011; Sheykhlouvand et al., 2018a), the values were converted to $\dot{V}$ O_2_ by multiplying by $\dot{V}$ O_2max_ in the same test before the calculation of the percent change, its standard error, and confidence interval. For $\dot{V}$ O_2max_, absolute (L·min^−1^) or relative changes normalized to body mass (mL·kg^−1^·min^−1^) were accepted; if both were available, we used the relative changes. Exercise economy was estimated from $\dot{V}$ O_2_ during running, rowing, or cycling at a submaximal velocity between 8 and 13.5 km h^−1^ and 75 and 220 W. Oxygen cost was converted to exercise economy.

**FIGURE 3:**
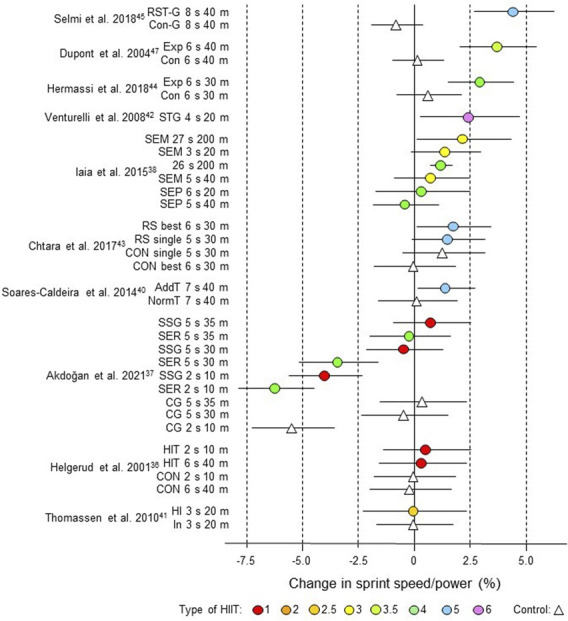
Forest plot of HIIT and control training effects on sprint speed/power for studies included in the meta-analysis. The data are study estimate scores representing percent changes in the HIIT (circles) and control (triangle) groups in descending order of the largest HIIT effect in each study. Error bars represent 90% confidence intervals. The type of HIIT is color- and number-coded, as shown in Figure 1. The abbreviations for the type of training are those of the original authors. RST-G, repeated-sprint training group; Con-G, control group; Exp, experimental group, Con, control group; STG, sprint-training group; SEM, speed endurance maintenance; SEP, speed endurance production; RS, repeated sprints; AddT; additional repeated-sprint training group; NormT, normal training group; SSG, small-sided games; SER; speed-endurance running; CG, control group; HIT, high-intensity training; HI, high intensity, IN, inactive.

**FIGURE 4:**
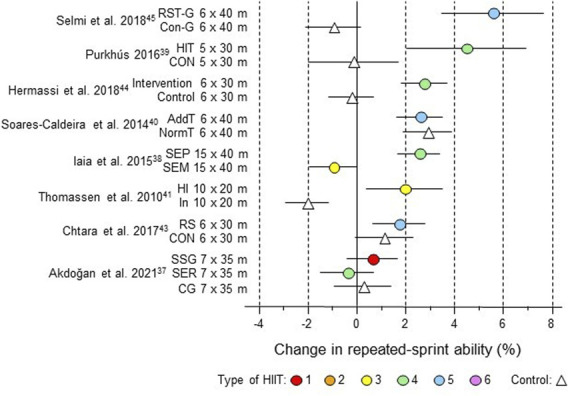
Forest plot of HIIT and control training effects on repeated-sprint ability for studies included in the meta-analysis. The data are study estimate scores representing percent changes in the HIIT (circles) and control (triangle) groups in descending order of the largest HIIT effect in each study. Error bars represent 90% confidence intervals. The type of HIIT is color- and number-coded, as shown in Figure 1. The abbreviations for the type of training are those of the original authors. RST, repeated-sprint training; Con-G, control group; HIT, high-intensity training; Con, control group; AddT; additional repeated-sprint training group; NormT, normal training group; SEM, speed endurance maintenance; SEP, speed endurance production; HI, high intensity, IN, inactive; RS, repeated sprints; SSG, small-sided games; SER, speed-endurance running; CG, control group.

**FIGURE 5:**
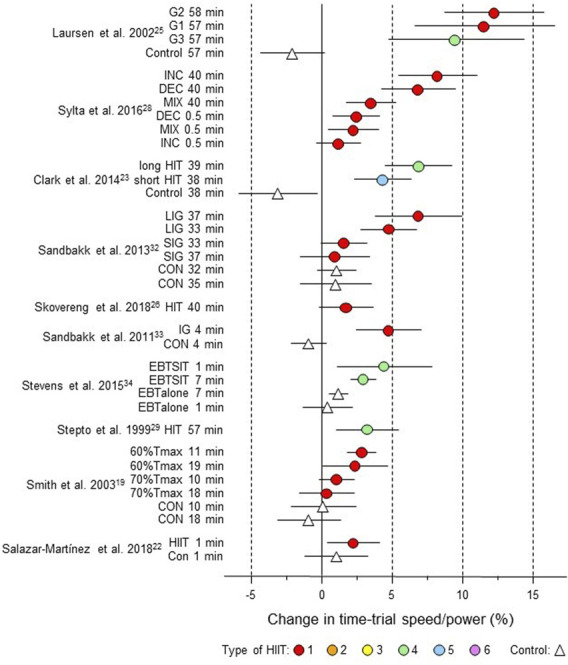
Forest plot of HIIT and control training effects on time-trial speed/power for studies included in the meta-analysis. The data are study estimate scores representing percent changes in the HIIT (circles) and control (triangle) groups in descending order of the largest HIIT effect in each study. Error bars represent 90% confidence intervals. The type of HIIT is color- and number-coded, as shown in Figure 1. The abbreviations for the type of training are those of the original authors. G1 to G3, group; INC, increasing HIT; DEC, decreasing HIIT; MIX; mixed HIT group; LIG, long-interval group; SIG, short-interval group; CON, control group; HIT, high-intensity training; IG, intervention group; EBTSIT, endurance-based sprint interval training; EBTalone, endurance-based training alone; T_max_, time for which $\dot{V}$ O_2max_ can be maintained.

**FIGURE 6:**
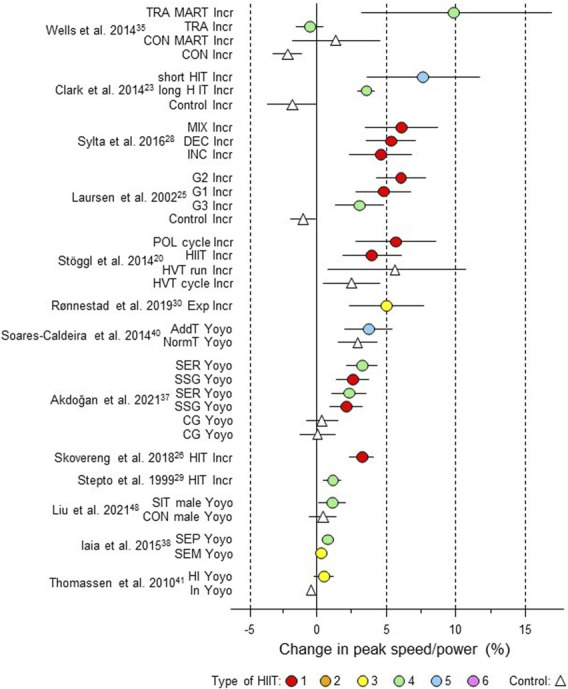
Forest plot of HIIT and control training effects on peak speed/power for studies included in the meta-analysis. The data are study estimate scores representing percent changes in the HIIT (circles) and control (triangle) groups in descending order of the largest HIIT effect in each study. Error bars represent 90% confidence intervals. The type of HIIT is color- and number-coded, as shown in Figure 1. The abbreviations for the type of training are those of the original authors. Incr, incremental; TRA MART Incr, training maximal aerobic running test incremental; TRA, training; HIT, high-intensity training; CON, control group; INC, increasing HIT; DEC, decreasing HIT; MIX; mixed HIT group; G1 to G3, group; POL, polarized; HVT, high-volume training; Exp, experimental; AddT; additional repeated-sprint training group; NormT, normal training group; SSG, small-sided game; SER; speed-endurance running; CG, control group; SIT, speed interval training; SEP, speed endurance production; SEM, speed endurance maintenance; HI, high intensity; IN, inactive.

**FIGURE 7:**
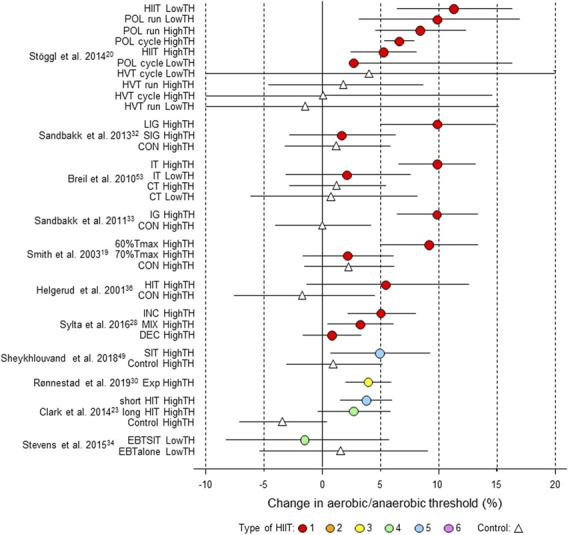
Forest plot of HIIT and control training effects on aerobic/anaerobic threshold for studies included in the meta-analysis. The data are study estimate scores representing percent changes in the HIIT (circles) and control (triangle) groups in descending order of the largest HIIT effect in each study. Error bars represent 90% confidence intervals. The type of HIIT is color- and number-coded, as shown in Figure 1. The abbreviations for the type of training are those of the original authors. TH, threshold; POL, polarized; LIG, long-interval group; SIG, short-interval group; IT, interval training; CT, control training; IG, intervention group; CON, control group; T_max_, time for which $\dot{V}$ O_2max_ can be maintained; INC, increasing HIT; DEC, decreasing HIT; MIX; mixed HIT group; EBTSIT, endurance-based sprint interval training; EBTalone, endurance-based training alone.

**FIGURE 8:**
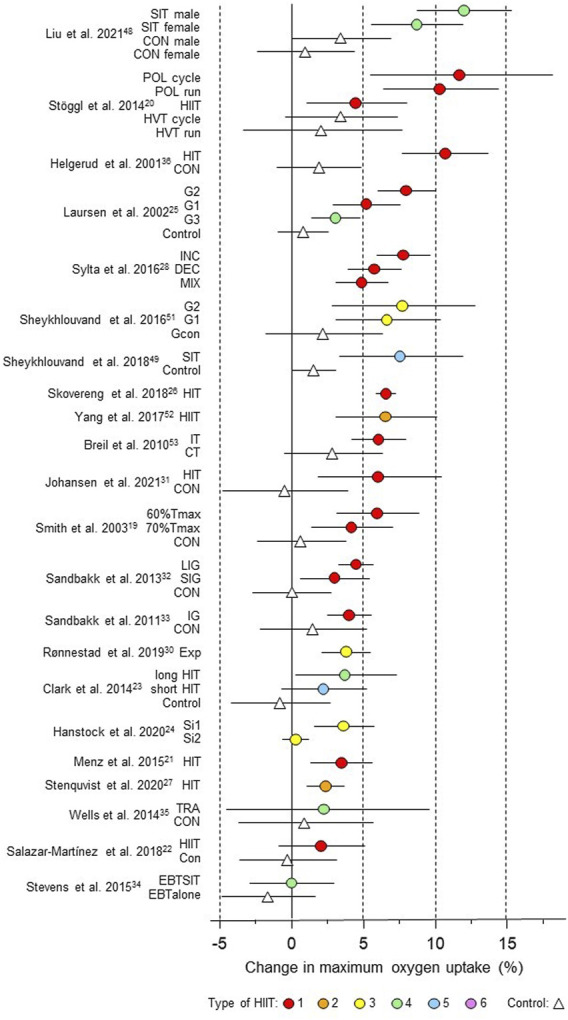
Forest plot of HIIT and control training effects on maximum oxygen uptake for studies included in the meta-analysis. The data are study estimate scores representing percent changes in the HIIT (circles) and control (triangle) groups in descending order of the largest HIIT effect in each study. Error bars represent 90% confidence intervals. The type of HIIT is color- and number-coded, as shown in Figure 1. The abbreviations for the type of training are those of the original authors. SIT, speed interval training; POL, polarized; HVT, high-volume training; HIT, high-intensity interval training; CON, control group; G1 to G3, group; INC, increasing HIT; DEC, decreasing HIT; MIX; mixed HIT group; IT, interval training; CT, control training; T_max_, time for which $\dot{V}$ O_2max_ can be maintained; LIG, long-interval group; SIG, short-interval group; SI, short intervals; TRA, training; EBTSIT, endurance-based sprint interval training; EBTalone, endurance-based training alone.

**FIGURE 9:**
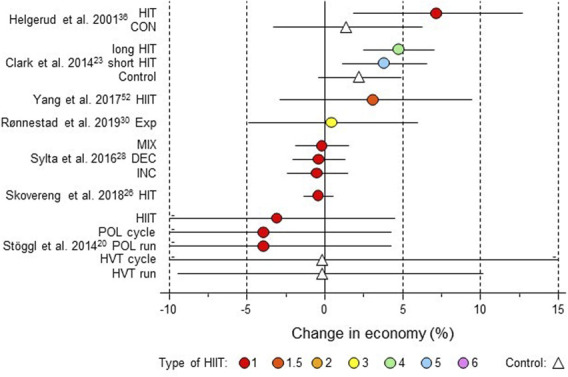
Forest plot of HIIT and control training effects on running or cycling economy for studies included in the meta-analysis. The data are study estimate scores representing percent changes in the HIIT (circles) and control (triangle) groups in descending order of the largest HIIT effect in each study. Error bars represent 90% confidence intervals. The type of HIIT is color- and number-coded, as shown in Figure 1. The abbreviations for the type of training are those of the original authors. HIT, high-intensity training; CON, control group; Exp, experimental group; INC, increasing HIT; DEC, decreasing HIT; MIX; mixed HIT group; POL, polarized; HVT, high-volume training.

The mean effects of HIIT and usual control training were calculated as percent changes. The standard error of the changes was calculated for each subgroup using *p*-values, t-values, or confidence intervals. Where these statistics lacked, the standard error was imputed via the typical error of measurement as follows (Carr et al., 2011; Vandenbogaerde and Hopkins, 2011): available standard errors were converted to typical errors and averaged separately (via variances weighted by degrees of freedom) for experimental and control groups for the given outcome variable; these typical errors were assumed to apply to experimental and control groups in those studies lacking standard errors; all typical errors were then converted to standard errors by multiplying by √2 and dividing by √(group sample size) (HIIT spreadsheet). Confidence limits for each study estimate shown in forest plots (Figures 3–9) and Supplementary Tables S3–S9 were calculated by log-transforming the estimates and their standard errors and then back-transforming to percent values.

### 2.5 Moderators

Tables 1–3 provide an overview of the sample size of the included studies and the moderators included in each meta-analysis. A few missing values of moderators were imputed, as shown in Supplementary Tables S1, S2. Nominal moderators were type of athlete (endurance or other), type of test (incremental or YoYo for peak speed/power), whether HIIT was performed as extra training or replacement, and phase of training, with values of 0, 0.5, or 1 for on-season, a mixture of on and off-season, and off-season. The type of HIIT was included as a numeric linear moderator [coded 1–6, thus from aerobic HIIT using classical intervals to SIT, as shown in Figure 1 and discussed in detail by Stöggl et al. (2024)]. Some studies performed a progressive HIIT training phase, for example, changing from HIIT type 1 of a long aerobic interval to HIIT type 2 of a short interval duration but higher intensity during the intervention period. For such studies, we included an average of two adjacent integers (e.g., 1.5). To account for the amount of training in each study, we used the weeks of training, which applied equally to the HIIT and control groups.

**TABLE 1:** Measures of the sample size of studies contributing to and excluded from the meta-analysis of each measure, with the list of moderators in the meta-analytic model.

	No. of studies Endur/other		No. of study estimates HIIT/Con	No. of imputed standard errors HIIT/Con	No. of participants: HIIT/Con; per study estimate mean ± SD	Maleness^a^HIIT/Con (fraction)	Moderators
**Sprint speed/power**	0/10		22/12	12/10	216/137; 10.4 ± 4.0	1/1	Intervention duration, type of HIIT^b^, log (pre-test time)^c^, log^2^ (pre-test time)^d^, and phase of training^e^
Excluded (female athletes)	2	0/1	1/1		8/8; 7.4 ± 0.5	0/0	
Excluded (arm test)		0/2	3/2		15/15; 8 ± 0	0.36/0.53	
**Repeated-sprint ability**	**0/8**		**10/7**	**4/3**	**102/79; 10.6 ± 3.3**	**0.90/0.86**	Maleness^a^, intervention duration, type of HIIT^b^, **pre-test decrement** ^f^, extra training^g^, and phase of training^e^
**Time-trial speed/power**	**9/0**		**19/6**	**0/0**	**320/69; 12 ± 10**	**1/1**	Intervention duration, type of HIIT^b^, **pre-test time** ^h^, extra training^g^, and phase of training^e^
Excluded (female athletes)	2/0		5/3		21/15; 7.2 ± 0.4	0.51/0.55	
Excluded (arm test)	1/0		1/1		8/8; 8 ± 0	1/1	
**Peak speed/power incremental**	**7/2**		**15/6**	**1/0**	**223/44; 13 ± 13**	**1/1**	Type of athlete^i^, intervention duration, type of HIIT^b^, type of testing^j^, **adjusted pre-test duration** ^k^, extra training^g^, and phase of training^e^
Excluded (females)	1/1		2/1		18/8; 8.7 ± 4.0	0.28/0.25	
**Peak speed/power YoYo**	**0/4**		**9/5**	**1/1**	**76/44; 8.6 ± 1.8**	**1/1**	
Excluded (female athletes)	3	0/2	3/3		31/30; 10.2 ± 2.0	0/0	
Excluded (outlier)		0/1	1/0		15/0; 15 ± 0	1/-	
**Anaerobic threshold**	**3/5**		**17/9**	**8/7**	**180/67; 9.5 ± 4.7**	**0.82/0.74**	Type of athlete^i^, maleness^a^, intervention duration, type of HIIT^b^, type of testing^l^, **pre-test threshold (% of** $\dot{V}$ **O**_**2max**_ **)** ^**m**^, and extra training^g^
**Aerobic threshold**	**2/1**		**5/4**	**1/1**	**41/24; 7.2 ± 3.0**	**0.91/0.74**	
$\dot{V}$ **O**_**2max**_	**15/7**		**34/17**	**7/6**	**394/132; 10.3 ± 8.2**	**0.87/0.80**	Type of athlete^i^, maleness^a^, intervention duration, type of HIIT^b^, **log(pre-test** $\dot{V}$ **O**_**2max**_ **)** ^c^ ^,^ ^n^, extra training^g^, and phase of training^e^
**Exercise economy**	**5/2**		**11/4**	**2/1**	**182/27; 14 ± 14**	**1/1**	**Pre-test intensity (% of** $\dot{V}$ **O**_**2max**_ **)**
Excluded (female athletes)	0/0		1/0		6/0; 6 ± 0	0.17/-	

Endur, endurance-trained athletes; other, non-endurance-trained athletes; HIIT, low-volume high-intensity interval training group; $\dot{V}$ O_2max_, maximum oxygen uptake; Con, control group.

Bold moderators represent the pre-test mean value appropriate for the meta-analysis. All moderators, except extra training and type of training, were interacted with treatment in our model.

The nature of the excluded data is shown in parentheses.

^a^ Proportion of male athletes; missing values were imputed to 1 (shown in parentheses).

^b^ Range of 1 (aerobic traditional long intervals) to 6 (anaerobic sprint intervals), as shown in Figure 1.

^c^ The need for log transformation was based on scatter plots of random-effect solutions.

^d^ The need for a quadratic term was based on scatter plots of random-effect solutions.

^e^ 0, during the competition phase; 1, outside the competition phase; and 0.5, mixture of competition and pre- or post-competition phase. Missing values were imputed to the mean of eligible study estimates (shown in parentheses).

^f^ Adjusted to the reported decrement of the original submission at the pre-test.

^g^ 0, HIIT partly or fully replaced conventional training; 1, HIIT was added to conventional training.

^h^ Adjusted to the time-trial time of the pre-test.

^i^ Endurance-trained athletes (e.g., cyclists, runners, and cross-country skiers) and non-endurance-trained athletes (e.g., ball sports and alpine skiing).

^j^ Incremental cycling or running tests or Yo-Yo tests.

^k^ The adjusted duration was the observed test duration multiplied by the mean fraction of the peak intensity in the test [test duration × (start intensity + end intensity)/2/end intensity)].

^l^ Speed or power at a low threshold (ventilatory threshold 1 or lactate threshold at ∼2) or high threshold (ventilatory threshold 2 or lactate threshold ∼4 mmol⸱L−1).

^m^ Percentage of $\dot{V}$ O_2max_ at ventilatory threshold 1 and 2 or lactate threshold at ∼2 or 4 mmol⸱L−1.

^n^ Pre-test mean $\dot{V}$ O_2max_ (mean of means ± SD of means ± mean of SDs) of endurance/other athletes: ♂64 ± 9 ± 6 (n = 28); ♀(n = 0); ♂50 ± 11 ± 22 (n = 10); and ♀41 ± 8 ± 7 (n = 4) mL∙kg−1∙min−1.

**TABLE 2:** Characteristics of training in the studies contributing to (in bold) and excluded from the meta-analysis of each measure.

	Phase of training^a^ Mean ± SD	Study duration (week) Mean ± SD; range	Training in the HIIT group		
			No. of sessions Mean ± SD; range	Extra training^b^ Mean ± SD	Type of HIIT^c^ Mean ± SD; range
**Sprint speed/power**	0.62 ± 0.49	5.6 ± 2.5; 2–12	15.2 ± 4.8; 9–24	0.06 ± 0.24	3.4 ± 1.5; 1–6
Excluded (female athletes) (n = 1)	0.0 ± 0.0	4.0 ± 0.0; 4–4	16.0 ± 0.0; 16–16	0.0 ± 0.0; 0	5.0 ± 0.0; 5–5
Excluded (arm test) (n = 2)	0.0 ± 0.0	3.3 ± 0.5; 3–4	11.3 ± 4.0; 9–16	0.0 ± 0.0; 0	3.7 ± 1.2; 3–5
**Repeated-sprint ability**	**0.64 ± 0.49**	**5.3 ± 1.9; 2–8**	**13.1 ± 3.7; 9–18**	**0.19 ± 0.40**	**3.8 ± 1.3; 1–5**
**Time-trial speed/power**	**0.50 ± 0.53**	**6.2 ± 3.6; 1–12**	**13.2 ± 7.6; 6–24**	**0.20 ± 0.41**	**1.8 ± 1.4; 1–5**
Excluded (female athletes) (n = 2)	0.0 ± 0.0	8.0 ± 0.0; 8–8	16.0 ± 0.0; 16–16	0.80 ± 0.45	1.0 ± 0.0; 1–1
Excluded (arm test) (n = 1)	1.0 ± 0.0	4.0 ± 0.0; 4–4	10.0 ± 0.0; 10–10	0.0 ± 0.0; 0	4.0 ± 0.0; 4–4
**Peak speed/power incremental**	**0.52 ± 0.46**	**6.2 ± 3.9; 0.86–12**	**15.0 ± 8.2; 6–28**	**0.33 ± 0.49**	**1.5 ± 0.5; 1–2**
Excluded (female athletes) (n = 1)	0.5 ± 0.0	4.3 ± 4.0; 2–9	15.0 ± 0.0; 15	0.0 ± 0.0	1.0 ± 0.0; 1–1
**Peak speed/power YoYo**	**0.29 ± 0.46**	**5.2 ± 2.0; 2–8**	**15.0 ± 5.4; 9–24**	**0.0 ± 0.0**	**1.7 ± 0.4; 1–5**
Excluded (females) (n = 2)	0.75 ± 0.46	8.0 ± 0.0; 8–8	18.0 ± 8.5; 12–24	0.50 ± 0.58	4.0 ± 0.0; 4–4
Excluded (outlier)^d^ (n = 1)	1.0 ± 0.0	7.0 ± 0.0; 7–7	14.0 ± 0.0; 14–14	1.0 ± 0.0	4.0 ± 0.0; 4–4
**Anaerobic threshold**	**0.37 ± 0.43**	**6.3 ± 3.6; 0.86–12**	**15.0 ± 8.2; 6–28**	**0.24 ± 0.44**	**1.8 ± 1.5; 1–5**
**Aerobic threshold**	**0.28 ± 0.26**	**6.3 ± 3.2; 2–9**	**16.6 ± 6.7; 10–28**	**0.0 ± 0.0**	**1.2 ± 0.4; 1–2**
$\dot{V}$ **O**_**2max**_	**0.30 ± 0.43**	**5.7 ± 3.1; 0.86–12**	**14.4 ± 6.2; 6–28**	**0.24 ± 0.43**	**2.1 ± 1.4; 1–5**
**Exercise economy**	**0.30 ± 0.48**	**7.2 ± 4.4; 0.86–12**	**17.0 ± 8.2; 6–28**	**0.53 ± 0.44**	**1.9 ± 1.5; 1–5**
Excluded (females) (n = 1)^e^	0.5 ± 0.0	9.0 ± 0.0; 9–9	15.0 ± 0.0; 15–15	0.0 ± 0.0	1.0 ± 0.0; 1–1

HIIT, low-volume high-intensity interval training group; n, number of studies excluded; $\dot{V}$ O_2max_, maximum oxygen uptake.

Studies of female athletes and arm tests were excluded when there were only one or two such studies, as shown in parentheses.

^a^ 0, during the competition phase; 1, outside the competition phase; and 0.5, mixture of competition and pre- or post-competition phase. Missing values were imputed to the mean of eligible study estimates (shown in parentheses).

^b^ 0, HIIT partly or fully replaced conventional training; 1, HIIT was added to conventional training.

^c^ Range of 1 (aerobic HIIT with traditional long intervals) to 6 (SIT: anaerobic sprint intervals), as shown in Figure 1.

^d^ Identified as an extreme value of the residual in the mixed model.

^e^ One study estimate, not the whole study, was excluded.

**TABLE 3:** Pre-test moderators for the different outcome measures of included studies. Data are the mean ± between-study SD (and ± mean of within-study SDs for $\dot{V}$ O_2max_).

	Sprint speed/power	Repeated-sprint ability	Time-trial speed/power	Peak speed/power	Aerobic/anaerobic threshold	$\dot{V}$ O_2max_	Exercise economy
Test duration							
Endurance male athletes (n = 25)			27 ± 21 min				
Other male athletes (n = 34)^a^	6.0 ± 5.4 s						
Adjusted duration (min)							
Endurance male athlete incr. test (n = 18)				8.4 ± 5.2			
Other male athlete incr. test (n = 4)				5.3 ± 3.5			
Other male athlete Yo-Yo test (n = 14)				7.4 ± 2.3			
RSA decrement (%)							
Other male athlete (n = 2)		4.9 ± 1.4					
Other female athlete (n = 15)		6.3 ± 1.1					
Threshold (% of $\dot{V}$ O_2max_)							
Endurance male athlete (n = 20)					75.0 ± 9.8		
Endurance female athlete (n = 7)^c^					75.9 ± 7.6		
Other male athlete (n = 2)					86.8 ± 0.9		
Other female athlete (n = 6)^b^					72.0 ± 13.5		
$\dot{V}$ O_2max_(ml∙kg^-1∙^min^-1^)^c^							
Endurance male athletes (n = 28)						62.8 ± 4.3 ± 6.0	
Endurance female athletes (n = 7)^d^						65.9 ± 3.5 ± 7.4	
Other male athletes (n = 10)						50.4 ± 9.3 ± 5.8	
Other female athletes (n = 6)^d^						44.6 ± 6.8 ± 3.8	
Intensity (% of VO_2max_)							
Endurance and other male athletes (n = 15)							69.8 ± 8.9

n, number of study estimates; incr., incremental; RSA, repeated-sprint ability; $\dot{V}$ O_2max_, maximum oxygen uptake.

^a^ The back-transformed mean of the log-transformed values was 4.8 ×∕÷ 1.9 s.

^b^ 42% endurance female athletes and 52% other female athletes.

^c^ The weighted back-transformed values of log-transformed $\dot{V}$ O_2max_ (ml∙kg−1∙min−1): 62.8 ×∕÷ 1.10 ×∕÷ 1.07 and 65.9 ×∕÷ 1.11 ×∕÷ 1.06 for endurance male and female athletes, respectively; 51.3 ×∕÷ 1.11 ×∕÷ 1.22 and 44.2 ×∕÷ 1.09 ×∕÷ 1.16 for other male and female athletes, respectively.

^d^ 45% endurance female athletes and 76% other female athletes.

Table 3 provides an overview of the pre-test moderators. Distinct physiological processes might determine sprint and time-trial performance of different distances and durations; therefore, we included pre-test time as a numeric linear moderator. For peak speed/power, adjusted test duration was included to account for the design of the incremental test, in which longer stages and smaller increments result in lower values (Morton, 1994; Luttikholt et al., 2006). To account for that, we adjusted the duration to that of a time trial in which the same amount of work was done using the formula: adjusted duration = (pre-test duration) × (pre-test mean speed/power)/(pre-test peak speed/power). For repeated-sprint ability, the reported percent decrement representing fatigue arising from the number and duration of sprints was included. For the aerobic/anaerobic threshold, the possible modifying effect of different threshold intensities was taken into account by including the pre-test threshold as a percent of $\dot{V}$ O_2max_. For $\dot{V}$ O_2max_, the inclusion of the pre-test mean accounted for possible ceiling effects on improvements. For exercise economy, the intensity at which economy was measured was $\dot{V}$ O_2_ expressed as a percent of $\dot{V}$ O_2max_; for some study estimates lacking $\dot{V}$ O_2_ (Skovereng et al., 2018; Sylta et al., 2016; Rønnestad and Vikmoen, 2019), it was estimated from the gross efficiency and workload by assuming the $\dot{V}$ O_2_ corresponding to a respiratory exchange ratio of 0.85 (Skovereng et al., 2018; Rønnestad and Vikmoen, 2019).

Apart from extra training and the type of HIIT, all moderators interacted with treatment. After initial explorations, a moderator that distinguishes between running and cycling performance was not included. The following interactions were investigated but were unclear and not included in the final models: type of HIIT with the duration of time trials on time-trial performance; type of HIIT with the duration of sprints on sprint performance; and type of HIIT with itself (a quadratic effect) on sprint performance. We investigated the number of HIIT sessions as an additional moderator, but it did not contribute usefully to the analysis owing to its strong correlations with weeks of training. There were insufficient study estimates to include sex as a moderator for sprint speed/power, time-trial speed/power, and peak speed/power. There were also insufficient study estimates to include arm tests for sprint speed/power or any moderators for exercise economy.

### 2.6 Data analysis

Random-effects meta-analysis models were realized using the mixed-model procedure (Proc Mixed). In each meta-analysis, the dependent variable was the log-transformed-extracted percentage change (100 × natural log[1 + percent change/100]). Sample estimates were weighted by the inverse square of their log-transformed standard errors, with the residual variance set to unity in the mixed model to perform the weighting (Rønnestad and Vikmoen, 2019; Yang, 2003). The fixed effects were treatment (HIIT or control) and the moderators described above. The random effects were study identity and sample-estimate identity within studies (with separate variances estimated for HIIT and control groups). The square root of the sum of study and sample-estimate variances provided an estimate of heterogeneity [the tau statistic (Higgins et al., 2024)] for HIIT and control treatments, representing differences between settings in predicted mean effects not due to sampling variation. The random effects were estimated, allowing only for positive variance, but standard errors of the variances were used to calculate appropriate lower and upper confidence limits, assuming a normal sampling distribution for the variances.

**FIGURE 10:**
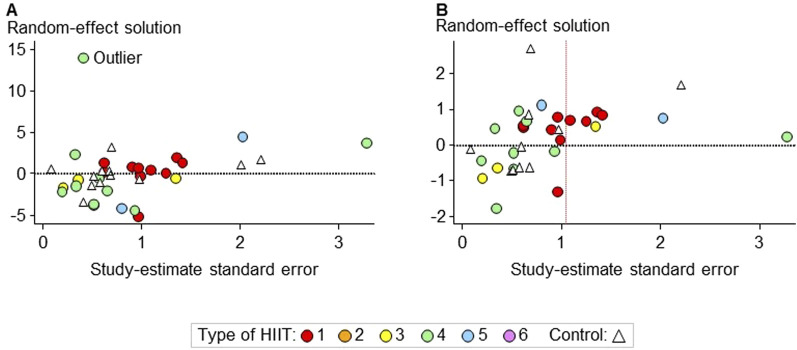
Scatterplot of the random-effect solution values versus the study estimate standard error before **(A)** and after **(B)** the exclusion of an extreme outlier in the meta-analysis of peak speed/power. The asymmetry in the scatterplot in **(B)**, represented by only positive values on the right to the red vertical dashed line, indicates possible publication bias. However, the mean bias of these seven values is trivial (<1%), and re-running the meta-analysis without the corresponding study estimates did not result in substantial changes in the predicted effects.

We examined scatter plots representing random-effect solution values versus standard errors of the study estimates to identify potential outliers and publication bias (Neville et al., 2022). One extreme outlier (Hermassi et al., 2018) was deleted in the peak speed/power analysis (shown in Supplementary Table S6). Potential publication bias was evident for peak speed/power (Figure 10A). However, running the analysis with the seven data points potentially contributing to publication bias (Figure 10B) did not result in substantial changes in the predicted effects. We retained these points to avoid loss of precision. To further address publication bias, we conducted simulations in SAS and RStudio to estimate any potential bias arising from the publication of only statistically significant effects on athlete endurance performance. Our analysis indicated that for the magnitudes of the effects observed, publication bias is expected to be negligible (Wiesinger et al., 2024a). Scatter plots of random-effect solution values versus linear predictors were examined for evidence that the use of simple linear moderators was adequate; the choice of log-transformation for some moderators (pre-test $\dot{V}$ O_2max_ for $\dot{V}$ O_2max_ and pre-test time for sprint speed/power) and the use of a quadratic term (test time for sprint speed/power) was motivated partly by these plots.

The effects of HIIT and control training were predicted for representative values of moderators for each meta-analysis, assuming that HIIT was performed during the competition phase and as replacements for some conventional training (see Tables 4–10 for details). The effects of the nominal moderators (e.g., male vs. female athletes, endurance vs. other athletes, and incremental vs. YoYo) are reported as differences between levels. The numeric moderators are evaluated as the effect of approximately two standard deviations (the mean of the pre-test study SDs of subject characteristics, such as $\dot{V}$ O_2max_, or the SD of study characteristics, such as study duration).

**TABLE 4:** Predicted effects, moderator effects, and heterogeneity SDs in the meta-analysis of sprint speed/power. Predicted effects are for 6 weeks of speed-endurance maintenance HIIT (aerobic rank = 3) and control training of other (non-endurance) male athletes in the on-season for 5- and 10-s leg tests and with HIIT performed as extra or replacement training.

	Effect (%) Mean; 90% CI	Magnitude of the observed effect^a^	Effect sign and probability^b^	Probability (%) ↓/↔/↑
Predicted effects				
5-s test; HIIT as extra training				
HIIT	3.3; 1.2–5.5	Moderate	↑***	0.2/4/96
Control	1.3; 0.1–2.5	Small	↔^0^↑*	0.5/34/66
HIIT–control	2.1; −0.3–4.4	Small	↑**	2/21/78
10-s test; HIIT as extra training				
HIIT	4.2; 2.1–6.4	**Moderate**	↑***	**0.0/0.9/99**
Control	−2.3; −5.3–0.8	**Small**	↓**	**80/16/4**
HIIT–control	6.7; 3.2–10.4	**Large**	↑***	**0.1/0.6/99**
5-s test; HIIT as replacement training				
HIIT	2.0; 1.1–2.9	**Small**	↑***	**0.0/4/95**
Control	1.3; 0.1–2.5	Small	↔^0^↑*	0.5/34/66
HIIT–control	0.7; −0.7–2.1	Trivial	↔	3/61/37
10-s test; HIIT as replacement training				
HIIT	2.9; 1.8–3.9	**Small**	↑****	**0.0/0.0/100**
Control	−2.3; −5.3–0.8	**Small**	↓**	**80/16/4**
HIIT–control	5.3; 2.1–8.7	Moderate	↑***	0.5/2/98
Moderator effects				
Type of HIIT (4.0 vs. 1.0)^d^	−0.4; −1.7–0.8	Trivial	↔	23/73/3
Test time 10 vs. 2.5-s HIIT^c^	2.9; 1.4–4.4	**Small**	↑***	**0.0/2/98**
Test time 10 vs. 2.5-s control^c^	−2.5; −6.3–1.5	Small	↓	77/17/7
Test time 10 vs. 2.5-s HIIT–control^c^	5.5; 1.3–9.9	Large	↑***	1/3/96
HIIT as extra training	1.3; −0.7–3.4	Small	↔^0^↑*	3/36/61
Duration +5-week HIIT	0.7; −0.6–2.0	Trivial	↔^0^↑*	2/65/33
Duration +5-week control	−0.9; −3.0–1.3	Trivial	↔	47/47/7
Duration +5-week HIIT–control	1.6; −0.7–4.0	Small	↔^0^↑*	4/29/67
On-season phase HIIT	2.6; 1.2–4.0	**Small**	↑***	**97/3/0.0**
On-season phase control	1.6; −0.2–3.4	Small	↑*↔^0^	72/27/2
On-season phase HIIT–control	1.0; −1.1–3.1	Trivial	↔	50/44/6
Heterogeneity SDs				
HIIT	1.2; 0.1–0.7	Small	↑**	3/6/92
Control	0.6; −0.9–1.2	Small	**↑**	20/24/56

CI, confidence interval.

^a^ Thresholds for small, moderate, large, very large, or extremely large increases: 1.0%, 3.0%, 5.5%, 8.6%, and 14%, respectively. Corresponding thresholds for decreases: 1.0%, −2.9%, −5.2%, −8.0%, and −12%.

^b^ Sign and probability are shown for effects with adequate precision at the 90% or 99% level.

↑↓ indicate substantial positive and negative effects, respectively; ↔ indicates trivial effects. Probabilities of substantial effects: *, possibly; **, likely; ***, very likely; and ****, most likely. Probabilities of trivial effects: ^0^, possibly; ^00^, likely; ^000^, very likely; and ^0000^, most likely. These probabilities are shown for effects with adequate precision (benefit/harm odds ratio >66 or chance of benefit <25%; for moderators and heterogeneity, chance of ↑ or ↓ <5%). Effects in bold have conservative adequate precision (chance of harm <0.1% or chance of benefit <5%; for moderators and heterogeneity, chance of ↑ or ↓ <0.5%).

^c^ The linear and quadratic coefficients are not shown. They were combined into the effect shown: mean ×∕÷ SD (5 s ×∕÷ 2).

^d^ Range of 1.0 (aerobic traditional long intervals) to 6.0 (anaerobic sprint intervals), as shown in Figure 1.

**TABLE 5:** Predicted effects, moderator effects, and heterogeneity SDs in the meta-analysis of repeated-sprint ability. Predicted effects are for 5 weeks of speed-endurance production HIIT (aerobic rank = 4) and control training of female and male other athletes in the on-season, with HIIT performed as replacement training and the RSA decrement of 5%.

	Effect (%) Mean; 90% CI	Magnitude of the observed effect^a^	Effect sign and probability^b^	Probability (%) ↓/↔/↑
Predicted effects^a^				
Male athletes (HIIT)	3.2; 1.3–5.1	Moderate	↑***	0.4/3/97
Male athletes (control)	−0.7; −5.5–4.4	Trivial	↓*↔^0^	44/35/22
Male athletes (HIIT–control)	3.9; −0.7–8.7	Moderate	↑**	4/8/88
Female athletes (HIIT)	−1.1; −6.8–4.9	Small		52/23/25
Female athletes (control)	−2.2; −11.3–7.9	Small	↓*	62/15/23
Female athletes (HIIT–control)	1.1; −7.9–11	Small		34/16/51
Moderator effects				
Male athletes–female athletes	2.8; −6.8–13.3	Small		24/13/63
Type of HIIT (4.0 vs. 1.0)^c^	−1.0; −4.5–2.7	Small	↓	50/35/15
HIIT as extra training	0.4; −3.5–4.5	Trivial		24/38/38
Duration +4-week HIIT	2.7; −0.8–6.4	Small	↑**	4/14/82
Duration +4-week control	2.4; −7.0–12.8	Small		20/15/65
Duration +4-week HIIT–control	0.3; −8.0–9.3	Trivial		38/20/43
On-season phase HIIT	1.2; −1.4–3.7	Small		56/37/7
On-season phase control	−1.1; −9.1–7.6	Small		28/21/51
On-season phase HIIT–control	2.2; −5.5–10.6	Small		64/17/20
RSAdec +3% HIIT	5.3; 1.0–9.7	Moderate	↑***	2/3/95
RSAdec +3% control	1.2; −6.2–9.1	Small		25/23/53
RSAdec +3% HIIT–control	4.0; −2.8–11.4	Moderate		10/11/80
Heterogeneity SDs				
HIIT	1.2; −1.0–2.0	Small		12/8/80
Control	2.3; −2.0–3.8	Moderate		16/2/81

CI, confidence interval.

^a^ Thresholds for small, moderate, large, very large, or extremely large increases: 1.0%, 3.0%, 5.5%, 8.6%, and 14%, respectively. Corresponding thresholds for decreases: 1.0%, −2.9%, −5.2%, −8.0%, and −12%.

^b^ Sign and probability are shown for effects with adequate precision at the 90% or 99% level.

↑↓ indicate substantial positive and negative effects, respectively; ↔ indicates trivial effects. Probabilities of substantial effects: *, possibly; **, likely; ***, very likely; and ****, most likely. Probabilities of trivial effects: ^0^, possibly; ^00^, likely; ^000^, very likely; and ^0000^, most likely. These probabilities are shown for effects with adequate precision (benefit/harm odds ratio >66 or chance of benefit <25%; for moderators and heterogeneity, chance of ↑ or ↓ <5%).

^c^ Range of 1.0 (aerobic traditional long intervals) to 6.0 (anaerobic sprint intervals), as shown in Figure 1.

**TABLE 6:** Predicted effects, moderator effects, and heterogeneity SDs in the meta-analysis of time-trial speed/power. Predicted effects are for 5 and 9 weeks of aerobic intermittent intervals HIIT (aerobic rank = 2) and control training of male endurance athletes mainly in the on-season, for 25- and 45-min tests with HIIT performed as replacement training and for leg tests only.

	Effect (%) Mean; 90% CI	Magnitude of the observed effect^a^	Effect sign and probability^b^	Probability (%) ↓/↔/↑
Predicted effects				
25-min test; 5-week duration				
HIIT	3.4; 2.2–5.5	**Moderate**	↑***	**0.1/0.7/99**
Control	0.5; −1.5–2.5	Trivial		10/56/34
HIIT–control	2.8; 0.7–5.0	**Small**	↑**	**0.4/7/93**
45-min test; 5-week duration				
HIIT	4.8; 3.2–6.4	**Moderate**	↑****	**0.0/0.1/100**
Control	−0.5; −3.2–2.2	Trivial	↓*↔^0^	38/45/17
HIIT–control	5.3; 2.2–8.6	Moderate	↑***	0.2/1/99
25-min test; 9-week duration				
HIIT	3.7; 1.2–6.3	Moderate	↑***	2/3/96
Control	4.0; −2.2–10.6	Moderate		9/12/79
HIIT–control	−0.3; −6.4–6.2	Trivial		42/21/36
45-min test; 9-week duration				
HIIT	5.1; 3.0–7.3	Moderate	↑***	0.2/0.6/99
Control	2.9; −3.8–10.1	Small		17/15/69
HIIT–control	2.1; −4.8–9.6	Small		22/17/61
Moderator effects				
Type of HIIT (4.0 vs. 1.0)^c^	0.4; −1.8–2.6	Trivial		14/56/30
Pre–test time +40-min HIIT	2.7; 0.9–4.5	**Small**	↑**	**0.2/6/94**
Pre–test time +40-min control	−2.1; −4.7–0.5	Small	↓**	80/17/3
Pre–test time +40-min HIIT–control	4.9; 2.1–7.8	**Moderate**	↑***	**0.1/1/99**
HIIT as extra training	1.8; −2.3–5.9	Small		12/25/64
Duration +6-week HIIT	0.5; −1.9–2.9	Trivial		11/58/31
Duration +6-week control	5.2; −1.7–12.6	Moderate		7/8/85
Duration +6-week HIIT – control	−4.5; −10.9–2.3	Moderate		81/10/9
Heterogeneity SDs				
HIIT	1.3; −1.0–2.1	Small		0.5/12/87
Control	0.7; −1.2–1.6	Small		25/15/60

CI, confidence interval.

^a^ Thresholds for small, moderate, large, very large, or extremely large increases: 1.0%, 3.0%, 5.5%, 8.6%, and 14%, respectively. Corresponding thresholds for decreases: 1.0%, −2.9%, −5.2%, −8.0%, and −12%.

^b^ Sign and probability are shown for effects with adequate precision at the 90% or 99% level.

↑↓ indicate substantial positive and negative effects, respectively; ↔ indicates trivial effects. Probabilities of substantial effects: *, possibly; **, likely; ***, very likely; and ****, most likely. Probabilities of trivial effects: ^0^, possibly; ^00^, likely; ^000^, very likely; and ^0000^, most likely. These probabilities are shown for effects with adequate precision (benefit/harm odds ratio >66 or chance of benefit <25%; for moderators and heterogeneity, chance of ↑ or ↓ <5%). Effects in bold have conservative adequate precision (chance of harm <0.1% or chance of benefit <5%; for moderators and heterogeneity, chance of ↑ or ↓ <0.5%).

^c^ Range of 1.0 (aerobic traditional long intervals) to 6.0 (anaerobic sprint intervals), as shown in Figure 1.

**TABLE 7:** Predicted effects, moderator effects, and heterogeneity SDs in the meta-analysis of peak speed/power. Predicted effects are for 6 weeks of speed endurance maintenance HIIT (aerobic rank = 3) and control training of male endurance and other athletes mostly for on-season, for 8-min incremental tests, and with HIIT performed as replacement training.

	Effect (%) Mean; 90% CI	Magnitude of the observed effect^a^	Effect sign and probability^b^	Probability (%) ↓/↔/↑
Predicted effects^c^				
Endurance incr. HIIT	3.4; 2.1–4.6	**Moderate**	↑****	**0.0/0.3/99.7**
Endurance incr. control	0.3; −1.7–2.3	Trivial		13/62/26
HIIT–control	3.1; 1.1–5.1	Moderate	↑***	0.3/4/95
Other incr. HIIT	0.0; −2.5–2.6	Trivial	↔^0^	24/51/25
Other incr. control	−1.3; −4.5–2.0	Small	↓*↔^0^	57/32/11
HIIT–control	1.3; −1.6–	Small		9/33/58
Other YoYo HIIT	1.7; 0.5–2.9	Small	↑**	0.2/14/86
Other YoYo control	0.5; −1.3–2.4	Trivial		8/60/33
HIIT–control	1.2; −0.6–3.0	Small		3/39/58
Moderator effects				
Type of HIIT (4.0 vs. 1.0)^d^	−0.3; −1.5–1.0	Small	↔^00^	14/82/5
Endurance–other for HIIT–control	1.7; −1.8–5.3	Small		10/26/64
YoYo–incr. for HIIT–control	−0.2; −3.3–3.2	Trivial		31/43/26
Extra training HIIT	1.2; −0.4 to 2.7	Small	↔^0^↑*	1/42/57
Duration +8-week HIIT incr.	1.0; −0.9–2.9	Trivial	↔^0^↑*	4/46/49
Duration +8-week control incr.	2.2; −2.7–7.3	Small		13/20/68
Duration +8-week HIIT–control incr.	−1.2; −6.0–3.9	Small		53/27/20
Test duration +8-min HIIT	0.4; −1.0–1.7	Trivial		5/75/20
Test duration +8-min control	−0.8; −3.7–2.1	Trivial		46/41/13
Test duration +8-min HIIT–control	1.2; −1.7–4.3	Small		10/35/56
Heterogeneity SDs				
HIIT	1.3; −0.5–1.9	Small	↑**	5/6/89
Control	1.8; −0.7–2.7	Moderate		6/3/91

CI, confidence interval. Incr, incremental.

^a^ Thresholds for small, moderate, large, very large, or extremely large increases: 1.0%, 3.0%, 5.5%, 8.6%, and 14%, respectively. Corresponding thresholds for decreases: 1.0%, −2.9%, −5.2%, −8.0%, and −12%.

^b^ Sign and probability are shown for effects with adequate precision at the 90% or 99% level.

↑↓ indicate substantial positive and negative effects, respectively; ↔ indicates trivial effects. Probabilities of substantial effects: *, possibly; **, likely; ***, very likely; and ****, most likely. Probabilities of trivial effects: ^0^, possibly; ^00^, likely; ^000^, very likely; and ^0000^, most likely. These probabilities are shown for effects with adequate precision (benefit/harm odds ratio >66 or chance of benefit <25%; for moderators and heterogeneity, chance of ↑ or ↓ <5%). Effects in bold have conservative adequate precision (chance of harm <0.1% or chance of benefit <5%; for moderators and heterogeneity, chance of ↑ or ↓ <0.5%).

^c^ Incr., incremental test; YoYo, YoYo intermittent recovery test.

^d^ Range of 1.0 (aerobic traditional long intervals) to 6.0 (anaerobic sprint intervals), as shown in Figure 1.

**TABLE 8:** Predicted effects, moderator effects, and heterogeneity SDs in the meta-analysis of aerobic/anaerobic threshold speed/power. Predicted effects are for 6 weeks of aerobic traditional long intervals HIIT (aerobic rank = 1) and control training of male and female endurance and other athletes mostly for off-season, with HIIT performed as replacement training and a pre-test intensity of 80% of $\dot{V}$ O_2max_.

	Effect (%) Mean; 90% CI	Magnitude of the observed effect^a^	Effect sign and probability^b^	Probability (%) ↓/↔/↑
Predicted effects				
Endurance male athletes (HIIT)	5.5; 3.0–8.0	**Large**	↑****	**0.0/0.4/99.6**
Endurance male athletes (control)	0.7; −1.8–3.3	Trivial		13/45/42
Endurance male athletes (HIIT–control)	4.8; 1.3–8.3	Moderate	↑***	0.4/3.2/96.4
Endurance female athletes (HIIT)	12.3; 4.9–20.2	Very large	↑***	0.4/0.5/99.1
Endurance female athletes (control)	−0.2; −7.6–7.6	Trivial		42/20/38
Endurance female athletes (HIIT–control)	12.6; 2.6–23.5	Very large	↑***	2/2/97
Other male athletes (HIIT)	6.1; 0.6–12.0	Large	↑**	2/5/94
Other male athletes (control)	1.0; −4.2–6.4	Trivial		25/26/50
Other male athletes (HIIT–control)	5.1; −2.1–12.8	Moderate		8/9/83
Other female athletes (HIIT)	7.6; 0.1–15.8	Large	↑**	3/4/93
Other female athletes (control)	1.4; −4.3–7.4	Small		22/23/55
Other female athletes (HIIT–control)	6.2; 2.5–15.7	Large		9/8/84
Moderator effects				
Endurance male athletes–female athletes	−6.9; −16.1–3.2	Large		85/6/9
Other male athletes–female athletes	−1.0; −12.8–12.3	Small		50/11/39
Female athlete endurance–other	6.0; −7.1–21.0	Large		19/8/74
Male athlete endurance–other	0.3; −7.9–7.9	Trivial		44/17/39
Type of HIIT (4.0 vs. 1.0)^c^	−2.7; −6.9–1.8	Small		75/17/8
Pre-test threshold +10% HIIT	−0.8; −2.2–0.7	Trivial	↓*↔^0^	27/68/5
Pre-test threshold +10% control	−0.7; −2.8–1.4	Trivial		41/50/9
Pre-test threshold +10% HIIT–control	0.0; −2.5–2.5	Trivial		20/51/30
HIIT as extra training	−1.6; −5.0–1.8	Small		63/27/10
Extra control training	−0.8; −6.2–4.9	trivial		48/26/27
HIIT–control	−0.8; −6.7–5.4	Trivial		48/22/30
Duration +7-week HIIT	−1,3; −4.5–2.0	Small		57/32/12
Duration +7-week control	2.1; −2.8–7.3	Small		14/21/65
Duration +7-week HIIT–control	−3.3; −8.7–2.3	Moderate		77/14/10
Heterogeneity SDs				
HIIT	2.3; −1.1–3.4	Moderate		8/2/90
Control	1.0; 2.5–2.9	Small		39/4/57

CI, confidence interval.

^a^ Thresholds for small, moderate, large, very large, or extremely large increases: 1.0%, 3.0%, 5.5%, 8.6%, and 14% respectively. Corresponding thresholds for decreases: 1.0%, −2.9%, −5.2%, −8.0%, and −12%.

^b^ Sign and probability are shown for effects with adequate precision at the 90% or 99% level.

↑↓ indicate substantial positive and negative effects, respectively; ↔ indicates trivial effects. Probabilities of substantial effects: *, possibly; **, likely; ***, very likely; and ****, most likely. Probabilities of trivial effects: ^0^, possibly; ^00^, likely; ^000^, very likely; and ^0000^, most likely. These probabilities are shown for effects with adequate precision (benefit/harm odds ratio >66 or chance of benefit <25%; for moderators and heterogeneity, chance of ↑ or ↓ <5%). Effects in bold have conservative adequate precision (chance of harm <0.1% or chance of benefit <5%; for moderators and heterogeneity, chance of ↑ or ↓ <0.5%).

^c^ Range of 1.0 (aerobic traditional long intervals) to 6.0 (anaerobic sprint intervals), as shown in Figure 1.

**TABLE 9:** Predicted effects, moderator effects, and heterogeneity SDs in the meta-analysis of maximum oxygen uptake. Predicted effects are for 5 weeks of aerobic traditional long-interval HIIT (aerobic rank = 1) and control training of male and female endurance and other athletes in the on-season, with HIIT performed as replacement training and separate mean pre-test $\dot{V}$ O_2max_ values^a^ for male and female endurance and other athletes.

	Effect (%) Mean; 90% CI	Magnitude of the observed effect^b^	Effect sign and probability^c^	Probability (%) ↓/↔/↑
Predicted effects				
Endurance male athletes (HIIT)	7.9; 5.9–9.9	**Large**	↑****	**0.0/0.0/100**
Endurance male athletes (control)	0.1; −1.8–2.1	Trivial	↔^0^	17/61/22
Endurance male athletes (HIIT–control)	7.7; 4.9–10.6	**Large**	↑****	**0.0/0.0/100**
Endurance female athletes (HIIT)	7.1; 3.3–10.9	**Large**	↑****	**0.0/0.0/100**
Endurance female athletes (control)	−0.3; −6.3–6.1	Trivial		58/30/12
Endurance female athletes (HIIT–control)	7.4; 0.2–15.1	Large	↑****	3/4/93
Other male athletes (HIIT)	11.1; 9.0–13.3	**Very large**	↑****	**0.0/0.0/100**
Other male athletes (control)	2.3; −0.4–4.3	Small	↑**	0.3/13/87
Other male athletes (HIIT–control)	8.6; 5.8 to 11.5	**Large**	↑****	**0.0/0.0/100**
Other female athletes (HIIT)	12.5; 9.0–16.1	**Very large**	↑****	**0.0/0.0/100**
Other female athletes (control)	0.9; −1.3 to 3.2	Trivial		10/45/47
Other female athletes (HIIT–control)	11.5; 7.4–15.8	**Very large**	↑****	**0.0/0.0/100**
Moderator effects				
Endurance male athletes–female athletes	−0.3; −5.6–6.6	Trivial		36/22/42
Other male athletes–female athletes	−2.6; −6.2–1.2	Small		76/18/6
Female athlete endurance–other	−3.7; −9.8–2.9	Moderate		76/13/12
Male athlete endurance–other	−0.8; −3.3–1.7	Trivial		46/43/12
Type of HIIT (4.0 vs. 1.0)^d^	−2.6; −4.1–−0.7	**Small**	↓***	**97/3/0.0**
Pre-test $\dot{V}$ O_2max_ +20% HIIT	−1.8; −3.1–−0.5	**Small**	↓**	**84/16/0.1**
Pre-test $\dot{V}$ O_2max_ +20% control	−0.4; −1.9–1.1	Trivial		25/69/6
Pre-test $\dot{V}$ O_2max_ +20% HIIT–control	−1.4; −3.3 to 0.6	Small	↓* ↔^0^	64/34/2
HIIT as extra training	1.1; −0.1–2.3	**Small**	**↔** ^**0**^ ↑*	**0.4/44/56**
Extra control training	−1,2; −3.6–1.3	Small		56/37/6
HIIT–control	2.3; −0.4–5.2	Small	↑**	2/19/79
Duration +5-week HIIT	1.2; 0.3–2.0	**Small**	**↔** ^**0**^ ↑*	**0.0/38/62**
Duration +5-week control	1,7; 0.1–3.6	Small	↑*	0.9/25/74
Duration +5-week HIIT–control	−0.6; −2.6–1.5	Trivial		36/54/10
On-season phase HIIT	3.6; 1.8–5.5	**Moderate**	↑***	**99.1/0.9/0.0**
On-season control	−0.6; −2.9–−1.6	Trivial		12/49/40
On-season phase HIIT–control	4.3; 1.4–7.2	**Moderate**	↑***	**97/3/0.2**
Heterogeneity SDs				
HIIT	1.1; −0.6–1.6	Small		6/11/83
Control	0.4; −0.8–1.0	Trivial		19/49/32

CI, confidence interval; $\dot{V}$ O_2max_, maximum oxygen uptake.

^a^ Effects are adjusted to the following values of moderators: pre-test $\dot{V}$ O_2max_, 63 mL kg-1∙min-1 for female and male endurance athletes and 41 and 51 mL kg-1∙min-1 for female and male other athletes.

^b^ Thresholds for small, moderate, large, very large, or extremely large increases: 1.0%, 3.0%, 5.5%, 8.6%, and 14%, respectively. Corresponding thresholds for decreases: 1.0%, −2.9%, −5.2%, −8.0%, and −12%. Thresholds for the random-effect SDs are ∼0.5 of these.

^c^ Sign and probability are shown for effects with adequate precision at the 90% or 99% level.

↑↓ indicate substantial positive and negative effects, respectively; ↔ indicates trivial effects. Probabilities of substantial effects: *, possibly; **, likely; ***, very likely; and ****, most likely. Probabilities of trivial effects: ^0^, possibly; ^00^, likely; ^000^, very likely; and ^0000^, most likely. These probabilities are shown for effects with adequate precision (benefit/harm odds ratio >66 or chance of benefit <25%; for moderators and heterogeneity, chance of ↑ or ↓ <5%). Effects in **bold** have conservative adequate precision (chance of harm <0.1% or chance of benefit <5%; for moderators and heterogeneity, chance of ↑ or ↓ <0.5%).

^d^ Range of 1.0 (aerobic traditional long intervals) to 6.0 (anaerobic sprint intervals), as shown in Figure 1.

**TABLE 10:** Predicted, moderator effects, and heterogeneity SDs in the meta-analysis of **exercise economy**. Predicted effects at an exercise-test intensity of 70% $\dot{V}$ O_2max_ for 7 weeks of HIIT and control training for male endurance and other athletes.

	Effect (%) Mean; 90% CI	Magnitude of the observed effect^a^	Effect sign and probability^b^	Probability (%) ↓/↔/↑
Predicted effects^a^				
HIIT	1.1; −0.5–2.8	Small		2/43/55
Control	1.5; −1.8–4.8	Small		10/30/60
HIIT–control	−0.3; −3.9–3.3	Trivial		38/37/26
Moderator effects				
Intensity +20% HIIT	0.3; −3.6–4.3	Trivial		29/35/37
Intensity +20% control	0.0; −6.1–6.5	Trivial		39/22/39
Intensity +20% HIIT–control	0.2; −6.9–7.9	Trivial		39/19/43
Heterogeneity SDs				
HIIT and control combined	2.2; −1.1–3.3	Moderate		9/2/89

CI, confidence interval.

^a^ Thresholds for small, moderate, large, very large, or extremely large increases: 1.0%, 3.0%, 5.5%, 8.6%, and 14%, respectively. Corresponding thresholds for decreases: 1.0%, −2.9%, −5.2%, −8.0%, and −12%.

^b^ Sign and probability are shown for effects with adequate precision (none for this measure).

### 2.7 Outcome statistics

The smallest important and other magnitude thresholds are derived from the variability in the performance of top athletes from competition to competition, which differs between sports (Malcata and Hopkins, 2014). We chose the smallest important value of ±1% for mean power in a competition performance, which is appropriate for cyclists, rowers, and kayakers and which is conservative for runners, whose smallest important difference is ±0.3%–±0.5% (Malcata and Hopkins, 2014). For the sprint performance of the other athletes, and presumably also for repeated sprints, the smallest important enhancement is ∼0.8% (Paton et al., 2001); we therefore applied the slightly more conservative threshold of 1%. The thresholds for small, moderate, large, very large, or extremely large increases are 0.3, 0.9, 1.6, 2.5, and 4.0 times the within-athlete variability in competition performance, respectively (Hopkins et al., 2009); applying these factors to log-transformed performance and back-transforming, the resulting thresholds for increases are 1.0%, 3.0%, 5.5%, 8.6%, and 14%, respectively, while those for decreases are −1.0%, −2.9%, −5.2%, −8.0%, and −12%, respectively.

The changes in laboratory and field tests that would result in the smallest and other enhancements in competition performance remain to be established. However, we assumed that percent changes in performance in these tests would directly transfer into percent changes in performance in a competition when the changes in performance in these tests are expressed as percent changes in power or its equivalent (Malcata and Hopkins, 2014). The transfer is obvious in sprints and time trials, provided that these are performed at intensities similar to competition intensities. Changes in peak power should transfer into changes in time trials performed at intensities similar to the peak power. The well-known physiological relationship that predicts endurance performance as the product of $\dot{V}$ O_2max_, exercise intensity as a percent of $\dot{V}$ O_2max_, and exercise economy (Joyner, 1991) implies that percent changes in each of these predictors translate directly into percent changes in time-trial speed/power provided that the other predictors do not change. Accordingly, changes in $\dot{V}$ O_2max_ should transfer into changes in endurance performance executed at intensities similar to $\dot{V}$ O_2max_, assuming no change in exercise economy; this transfer should also apply to longer-endurance performances executed at sub- $\dot{V}$ O_2max_ intensities. Changes in aerobic/anaerobic threshold performance and exercise economy should also transfer into endurance performance executed at intensities similar to those of the tests. All these assumptions apply to the performance of endurance athletes, but for some of the measures ( $\dot{V}$ O_2max_, peak power, and threshold), there were sufficient data to estimate effects on other athletes (ball or racket sports and alpine skiing); for these effects and athletes, we assumed the same magnitude thresholds as for endurance athletes, but it remains to be determined how percent changes in the tests transfer to changes in competition performance.

Sampling uncertainty of the estimates from each meta-analysis is presented as ± 90% confidence limits and as quantitative chances of substantial and trivial magnitudes based on a Bayesian analysis with a minimally informative prior (Hopkins, 2022). A probability (chances/100) and its complement (1 minus the probability) are *p*-values for tests of the hypotheses that the effect has the given magnitude and does not have the given magnitude, respectively (Hopkins, 2022). An effect on the predicted mean performance was deemed to have adequate precision if it was potentially implementable (benefit/harm odds ratio >66) or was unlikely to be beneficial (chance of benefit <25%). Mean effects with conservative, adequate precision had either a probability of harm <0.1% or a probability of benefit <5% and are highlighted in bold in tables; up to five of the former independent effects could be implemented while limiting the overall risk of harm to the most unlikely (<0.5%), while up to five of the latter could not be implemented while limiting the overall probability of benefit to the unlikely (<25%). Moderator effects and heterogeneity SDs were deemed to have adequate precision if at least one substantial hypothesis was rejected at the 5% level (probability of a substantial magnitude <5% or very unlikely). Adequate precision was conservative if the hypotheses were rejected at the 0.5% level (probability of a substantial magnitude <0.5% or most unlikely) and are highlighted in bold in tables; the overall error rate for rejecting the substantial hypotheses with up to 10 such independent effects is controlled to <5%. For effects with adequate precision, probabilities of substantial and/or trivial magnitudes >25% were interpreted qualitatively using the following scale: 25%–75% indicates possible, some, or modest evidence; 75%–95% indicates likely or good evidence; 95%–99.5% indicates very likely, very good evidence, and; >99.5% indicates most likely, strong evidence (Hopkins, 2022).

## 3 Results

From an initial pool of 17,176 records, 34 articles met the inclusion criteria, and their descriptive statistics are provided in Supplementary Tables S3–S9. The study quality score was 9.7 ± 0.9 (mean ± SD; range, 6.7–10.9; Supplementary Tables S1, S2). Tables 1–3 summarize sample size, training measures, and moderators in the meta-analysis of each outcome measure. The spreadsheet of all data imported into SAS is available under the following link (HIIT spreadsheet).

### 3.1 Mean effects of HIIT

**FIGURE 11:**
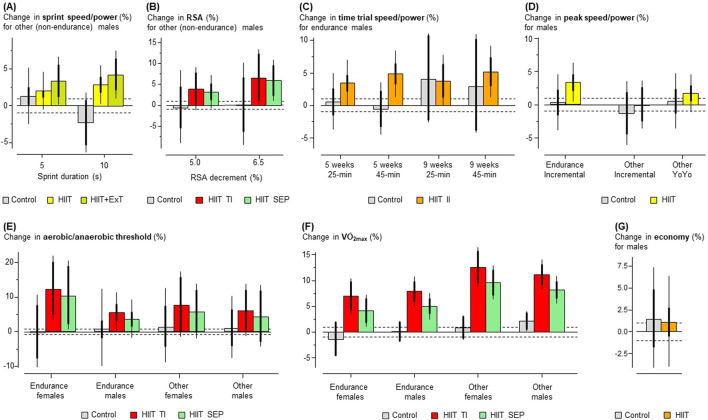
Predicted mean percent changes in the seven outcome measures with HIIT and control training for some practically important values of moderators. Thick and thin error bars represent 90% confidence intervals for mean and individual settings, respectively. Dotted lines at ±1% represent the smallest important changes. $\dot{V}$ O_2max_, maximum oxygen uptake; HIIT, high-intensity interval training; +ExT, extra training; TI, traditional interval; SEP, speed endurance production; II, intermittent interval; YoYo, intermittent recovery running tests.

Figure 11 shows a concise summary of the predicted mean effects of HIIT and control training across all performance measures in settings defined by some practically important values of moderators. All measures, except exercise economy, showed clear substantial improvements in at least one setting, considering the uncertainties in the mean effects represented by thick error bars. For male endurance athletes, these clear effects were ∼5%. Effects for female athletes, where they could be estimated (aerobic/anaerobic threshold and $\dot{V}$ O_2max_), tended to be greater than those for male athletes (∼10%). The enhancements in the team-sport-relevant performance measures (sprint and repeated-sprint ability) were similar to the enhancements in the other performance measures among male athletes. However, other athletes tended to experience greater improvements than endurance athletes in $\dot{V}$ O_2max_ and less improvement in the aerobic/anaerobic threshold and peak speed/power. Mean changes in performance with control training were generally less than with HIIT, and given their uncertainties, all true effects could be trivial.

Tables 4–10 complement the predicted HIIT effects summarized in Figure 11, adding detailed statistics for all subgroup comparisons, estimable moderator effects, and heterogeneity (random effect) SDs in the meta-analyses of each performance measure. Inferential comparisons of HIIT with control training confirm the effects of HIIT mentioned above.

For male endurance athletes, there was strong evidence for an increase in $\dot{V}$ O_2max_ (7.7%) and good to very good evidence for substantial improvements in time-trial speed/power, peak speed/power, and aerobic/anaerobic threshold (3.1%–5.3%). For female endurance athletes, the aerobic/anaerobic threshold and $\dot{V}$ O_2max_ showed good and strong evidence for increases (12.6% and 7.4%, respectively).

For male athletes, the effect of HIIT on sprint speed/power in a 5-s lasting sprint test was unclear when it replaced some conventional training, but the evidence for enhancements with 5 and 10-s sprint tests was otherwise good or very good (3.9%–6.7%). For these athletes, strong evidence and good evidence for enhancements were found for changes in $\dot{V}$ O_2max_ and repeated-sprint ability (8.6% and 3.9%, respectively), but the changes in the peak power and aerobic/anaerobic threshold were unclear. There was strong evidence of enhancement in $\dot{V}$ O_2max_ for female athletes (11.5%), while effects were unclear for other outcomes for these athletes (repeated-sprint ability and aerobic/anaerobic threshold).

### 3.2 Moderator effects

#### 3.2.1 Differences between groups

The detailed moderator effects for each outcome measure are shown in Tables 4–9, with a summarized overview in Supplementary Table S10. Where they could be estimated, moderating effects representing differences between groups of athletes and tests, including type of athlete (endurance vs. other), biological sex (female vs. male), and type of test (incremental vs. YoYo for peak speed/power), were inconclusive.

#### 3.2.2 Type of HIIT

We analyzed the moderating effect of the type of HIIT on each measure by comparing predicted differences in the effect of SEPT, coded as 4, and aerobic HIIT with traditional long intervals, coded as 1. There was very good evidence of a negative effect of more anaerobic types of HIIT on $\dot{V}$ O_2max_ (−2.6%) and good evidence of a trivial effect (weak evidence for a negative effect) on peak speed/power (−0.3%). For the remaining measures, the moderating effect of the type of HIIT was either unclear (sprint speed/power, repeated-sprint ability, time-trial speed/power, and aerobic/anaerobic threshold) or could not be estimated (exercise economy).

#### 3.2.3 Duration of HIIT intervention

The predicted effect of increasing the weeks of intervention duration by ∼2 SDs ranged from increases for repeated-sprint ability, $\dot{V}$ O_2max_, peak speed/power, and sprint speed/power (2.7%–0.7%; good to modest evidence) to unclear for time-trial speed/power and a decrease for the aerobic/anaerobic threshold (−1.3%; good evidence). Increased intervention duration for the control group had unclear effects for all measures except $\dot{V}$ O_2max_, where the evidence for an increase was modest (1.7%).

#### 3.2.4 HIIT in different training phases

Where it could be estimated, there was very good evidence for additional beneficial effects of HIIT in the on-season for sprint speed/power (2.6%) and $\dot{V}$ O_2max_ (3.6%); for $\dot{V}$ O_2max_, the effect in the HIIT groups compared with the unclear effect in control remained clearly beneficial (4.3%). Training during the on-season had a small observed beneficial effect for the HIIT group alone and in comparison to the control, but these effects were unclear.

#### 3.2.5 HIIT as replacement or additional training

The effects of increasing the training volume by adding HIIT to conventional training were unclear in the HIIT groups for repeated-sprint ability, time-trial speed/power, and aerobic/anaerobic threshold. There was some evidence for additional benefits of extra training for sprint speed/power (1.3%), peak speed/power (1.2%), and $\dot{V}$ O_2max_ (1.1%); there was good evidence of an increase in $\dot{V}$ O_2max_ compared with the control (2.3%).

#### 3.2.6 Pre-test moderators

Increasing the performance-test duration by ∼2 SDs had similar effects for sprint (10 vs. 2.5 s) and time-trial speed/power (+40 min), i.e., ∼3% for HIIT, ∼−2% for control, and ∼5% for the comparison of HIIT and control, with evidence for substantial effects ranging from good to very good. For peak speed/power (+8 min), the same pattern was observed, showing a greater effect for HIIT and a lower effect for conventional training in longer incremental or YoYo tests: 0.4% for HIIT, −0.8% for control, and 1.2% for comparison of HIIT and control. There was very good evidence for more improvement with HIIT (5.3%) when the repeated-sprint ability test produced more fatigue (RSA decrement +3%), but the moderating effect of fatigue was unclear in the control group and in the comparison of HIIT with the control. There was a possibility of less effect of HIIT (−0.8%) on the speed/power threshold at higher-threshold intensities (percent of $\dot{V}$ O_2max_ + 10%), but the effects in the control group and in the comparison with HIIT were unclear. Higher pre-test values of $\dot{V}$ O_2max_ (mean $\dot{V}$ O_2max_ + 20%) showed good evidence of reduced effects of HIIT on $\dot{V}$ O_2max_ (−1.8%) and some evidence (−1.4%) when compared with the unclear effect in the control group. Finally, the intensity at which exercise economy ( $\dot{V}$ O_2_ expressed as percent of $\dot{V}$ O_2max_ + 20%) was measured had unclear effects in both groups and when the groups were compared.

### 3.3 Heterogeneity

Standard deviations representing heterogeneity in the effects of HIIT and control training between studies had adequate precision only for sprint and peak speed/power in the HIIT group, both of small magnitude with good evidence of substantial. Observed magnitudes otherwise ranged from moderate (one for HIIT and one for control) to trivial (one for control), with a suggestion of greater heterogeneity in the effects of HIIT.

Heterogeneity and its uncertainty add to the mean effects and their uncertainty to yield the prediction intervals for effects in specific new settings, as shown in Figure 11. The prediction interval is only slightly wider than the confidence interval of the mean effect for $\dot{V}$ O_2max_ and time-trial speed/power after 9 weeks of HIIT, but for most other measures, there is considerable widening due to heterogeneity, often a doubling of width.

### 3.4 Errors of measurement

These are summarized in Supplementary Table S10 as observed SDs in HIIT and control groups averaged across all studies in which they could be estimated. The means range from 1.4% to 3.5% in HIIT groups and 1.3%–4.4% in controls. The means for the HIIT groups are either similar to or greater than those of controls. Standard deviations representing individual responses to HIIT beyond those occurring with control training were derived from the errors of measurement and are also shown in Supplementary Table. SDs range from ∼1% to ∼7%.

## 4 Discussion

We conducted meta-analyses on seven performance-related measures in various subgroups of highly trained endurance and other elite athletes with different types of HIIT. The measures and predictors of performance were sprint speed/power, repeated-sprint ability, time-trial speed/power, peak speed power, aerobic/anaerobic threshold, $\dot{V}$ O_2max_, and exercise economy. We found substantial improvements in performance for all outcomes when HIIT was compared with conventional (control) training, except for exercise economy, but comparisons of female and male endurance and non-endurance athletes were inconclusive. The type of HIIT was a substantial moderator for $\dot{V}$ O_2max_ such that greater improvements were seen with longer intervals and lower intensities. These results have useful implications for researchers, athletes, and coaches, especially when considering our findings with some effect moderators.

### 4.1 Mean effects of HIIT

The magnitude of the effects varies across the predictors and measures of performance among elite endurance and other athletes. The biggest effects of HIIT on $\dot{V}$ O_2max_ appeared to be greater than those on time-trial performances (Tables 6, 9). Formal inferential comparison of the effects on the various measures of performance is challenging because of differing moderator values and dependent-variable interdependence. Nevertheless, inspection of their confidence intervals indicates that the greater effect on $\dot{V}$ O_2max_ is not simply due to sampling uncertainty. It follows from the physiology of endurance performance (Joyner, 1991) that these greater improvements in $\dot{V}$ O_2max_ were accompanied by a reduction in either or both of the fractional utilization of $\dot{V}$ O_2max_ (and probably, therefore, aerobic/anaerobic threshold) and exercise economy. However, we found similar effects of HIIT on the aerobic/anaerobic threshold as time-trial speed/power and a trivial (but unclear) effect of HIIT on exercise economy. This apparent discrepancy may arise from including different study estimates and moderator selections for each outcome measure but underscores the need for mediation analysis, which is presented via relationships between the changes in these different predictors of endurance performance by Wiesinger et al. (2024b).

### 4.2 Moderator effects

The moderator effects impact the implementation of HIIT in real-world sports, exploring the following key considerations: differences between the types of athlete and test; the type of HIIT; the optimal duration of HIIT intervention; the phase of implementing HIIT within the annual training cycle; and whether HIIT should augment or replace some conventional training.

#### 4.2.1 Differences between groups

More studies are required to resolve the unclear modifying effects of sex (female vs. male), type of athlete (endurance vs. other), and type of test (incremental vs. YoYo). Any modifying effects that turn out to be clearly substantial will be easily explained by differences in physiology and/or differences in the headroom for improvement between these types of athletes (Ansdell et al., 2020). The strength of evidence for the difference between these groups is, in any case, largely immaterial because HIIT has substantial effects on most measures of performance for each of the groups.

#### 4.2.2 Type of HIIT

Defining six types of HIIT as a graded continuum (Stöggl et al., 2024) enabled us to include this potential moderator in the meta-analyses. Disappointingly, the modifying effect was clear only for one measure, $\dot{V}$ O_2max_, such that the more aerobic the HIIT, the greater the improvement. The effect on the aerobic/anaerobic threshold was of similar magnitude but unclear, and the observed effects on the other measures of endurance performance (including especially time-trial speed/power) were trivial and inferentially either unclear or likely to be trivial. It would therefore be premature to recommend a particular type of HIIT for endurance athletes, although Wiesinger et al. (2024b) showed that an analysis of the relationships between changes in some of the measures suggests that training with a combination of HIIT types might be a useful strategy. The modifying effects of the type of HIIT on sprints and repeated sprints were also unclear, with observed trivial and marginally small magnitudes favoring more aerobic HIIT, but a recommendation for the best type of HIIT for team-sport athletes would also be premature.

#### 4.2.3 Duration of HIIT intervention

How long should a HIIT program be implemented? For all but the aerobic/anaerobic threshold, there were trivial to small beneficial modifying effects of additional two SDs of weeks of training, although the evidence was good only for repeated-sprint ability. Inspection of residuals vs. study duration did not provide any obvious evidence for a non-linear effect consistent with a plateau in the performance change at the longer durations, so it would seem that small additional improvements might accrue from extending HIIT beyond the durations in the published studies. There was a tendency (albeit mostly unclear) with the endurance measures toward control groups “catching up” with the HIIT groups over longer durations. Whether it would be better to continue with HIIT (which could lead to staleness-related erosion of the improvements in endurance performance) or to eventually switch from HIIT to usual or some other training (including a different type of HIIT), therefore, cannot be determined without more research that includes longer durations and/or other periodization strategies (Hermassi et al., 2018).

#### 4.2.4 HIIT during different parts of the season

Does the effectiveness of HIIT depend on which phase of the season it is implemented? This practical question was not addressed in previous meta-analyses (Gist et al., 2014; Sloth et al., 2013; Bacon et al., 2013; Weston et al., 2014; Milanovic et al., 2015; Carr et al., 2011), and we compared the implementation in only on-season with off-season (an aggregate of off-season, general preparation, and pre-competition). We found very good evidence for greater enhancement when HIIT was implemented in the on-season for sprint speed/power and $\dot{V}$ O_2max_ (for $\dot{V}$ O_2max_ also for HIIT relative to control), but the effect was unclear for repeated-sprint ability and could not be estimated for other performance measures. The greater on-season effect of HIIT on sprint speed/power and $\dot{V}$ O_2max_ is presumably driven by different adaptations in metabolic pathways—predominantly anaerobic pathways for sprint speed/power (Bangsbo et al., 1994; Rodas et al., 2000) and predominantly aerobic pathways for $\dot{V}$ O_2max_ (Fox et al., 1973; Bassett and Howley, 2000). Given that repeated-sprint performance is mediated by anaerobic and aerobic pathways (Girard et al., 2011; Nevill et al., 1989; Bishop and Edge, 2006; Bishop et al., 2004), one could expect a greater benefit from HIIT for this measure in the on-season, and the uncertainty in the unclear effect allows for this outcome. These findings challenge the traditional expectation that there are more opportunities to improve the performance during the off-season, but more studies with better demarcation of season phases are needed to determine which phase is best for the implementation of HIIT and whether it should be implemented across phases.

#### 4.2.5 HIIT as replacement or additional training

Whether HIIT should augment or partially replace conventional training is another important practical issue. Only $\dot{V}$ O_2max_ and aerobic/anaerobic threshold had data from control groups that received extra control training, and only $\dot{V}$ O_2max_ had adequate precision, with good evidence favoring augmented training. This finding is contrary to our expectation that highly trained athletes are at a near-optimum level of training in the on-season and, therefore, that any extra training could be counterproductive (of which there was a tendency in the control group). Of the remaining measures, there was only some evidence for augmented HIIT with sprint speed power (other athletes) and peak speed/power (endurance athletes), while the observed benefit of augmented HIIT with time-trial speed/power was unclear. Together, these results are consistent with headroom for improvement with HIIT for highly trained endurance and other elite athletes in the on-season (Reilly, 2006; Jeong et al., 2011), but more evidence is needed to address the possibility that augmented conventional training is equally effective or even detrimental for most measures of performance. This discussion is limited by the lack of information on the specific nature of the usual control training regimens (see Limitations). However, it is reasonable to assume that control training protocols varied across studies, depending on the sport, training culture, and regional differences. These variations could influence the observed effectiveness of HIIT on performance measures and predictors in different ways.

#### 4.2.6 Pre-test moderators

For $\dot{V}$ O_2max_, the pre-test moderator addresses the extent of different effects of HIIT in study settings with different baseline mean values. The modest evidence of a negative effect (less effect of HIIT with higher baseline $\dot{V}$ O_2max_) is consistent with the notion that the more highly trained the athletes, the less the headroom for improvement in performance (Laursen and Jenkins, 2002; Midgley et al., 2007; Scheuer and Tipton, 1977). Given the magnitude of this modifying effect, athletes with $\dot{V}$ O_2max_ one or two SDs above their group mean will still gain a clear benefit with HIIT. However, the estimate of the moderating effect represents an average over male and female endurance and other athletes. With more study estimates, an interaction of pre-test $\dot{V}$ O_2max_ with the athlete group would determine whether the magnitude differs between these four types of athletes.

The pre-test moderators for the other performance measures each represent an aspect of the load or intensity of the performance test. There is too much uncertainty in the moderating effect of submaximal intensity on HIIT’s effect on the exercise economy for any useful conclusion beyond the need for more studies. The moderating effect of the intensity of the anaerobic threshold on threshold speed/power was likewise poorly defined, with only some evidence of a negative effect in the HIIT groups. HIIT was clearly more effective with longer sprints and longer time trials, which suggests that HIIT has more effects on the aerobic than anaerobic components of performance. There was an opposite modifying effect of duration with control training (unclear for sprints and good evidence for time trials), which may have been due to a greater nocebo effect with longer and, therefore, harder tests, at least for time trials. The duration of the peak speed/power test had similar contrasting but smaller and less well-defined modifying effects on HIIT and control training. The two SDs of duration for evaluating the effect in this test were only +8 min, but when evaluated over the duration used for the time trial (+40 min), the effects in the HIIT and control groups would be similar to those in the time trial.

Repeated-sprint tests with a greater decrement in performance over the duration of the test must have been more intense, and these also showed clearly greater effects with HIIT. Unfortunately, greater uncertainty in a smaller moderating effect with control training made the net effect unclear, but it seems reasonable to posit that HIIT’s effect on the aerobic system ( $\dot{V}$ O_2max_) produced a greater enhancement in the more intense tests by reducing fatigue. This explanation is consistent with a positive association between repeated-sprint ability and $\dot{V}$ O_2max_ (Girard et al., 2011; Nevill et al., 1989; Bishop and Edge, 2006; Bishop et al., 2004).

### 4.3 Heterogeneity

Real differences between the effects of HIIT, characterized as SDs presenting between-study heterogeneity, were almost all substantial but estimated with inadequate precision. A comparison of heterogeneity for HIIT vs. control was therefore not attempted, but the observed tendency toward greater heterogeneity for HIIT is the expected result since there is likely to be more variation in the HIIT programs and, consequently, in their effects than in controls, even after adjustment for the known subject and study moderators.

Figure 11 shows that heterogeneity and its uncertainty have added substantially to the uncertainty in a mean effect for most measures and thereby weaken the strength of evidence for the effect of HIIT in an individual study setting. For some measures and mean settings (e.g., $\dot{V}$ O_2max_ for male and female endurance and other athletes), the 90% prediction intervals fall entirely in beneficial values, so HIIT is effective in at least 95% of individual settings. However, for some measures, HIIT could be ineffective or even harmful in a substantial proportion of individual settings, especially when the prediction interval in control training is taken into account. The most important measure for endurance athletes is time-trial speed power, and for other athletes, the most important measures are sprint speed/power and repeated-sprint ability. For these measures of performance, Figure 11 shows that there are individual settings where HIIT does better than control training, but control training could be equally effective, if not better, in other individual settings. We, therefore, cannot recommend HIIT unprovisionally for implementation in individual settings unless and until heterogeneity and its uncertainty are reduced sufficiently in an updated meta-analysis with more studies.

### 4.4 Errors of measurement

The mean errors in the control groups are similar to, or a little larger than, those in short-term reliability studies for trained individuals (Hopkins et al., 2001), implying relatively little contribution of individual responses to error of measurement with control training over the duration of the studies. Larger errors of measurement in the HIIT groups (for sprint speed/power, repeated-sprint ability, time-trial speed/power, peak speed/power, and exercise economy) reflect substantial contributions of individual responses to HIIT (SDs of up to 6.8%), but there is bound to be large uncertainty for these estimates arising from sampling variation. Sampling variation is also likely responsible for the lower errors of measurement with HIIT than with control training for aerobic/anaerobic threshold and $\dot{V}$ O_2max_ (resulting in negative individual-response SDs) because we would not expect HIIT to have fewer individual responses than control training with these two measures.

### 4.5 Publication bias

Although we found little evidence of publication bias across all the measures, our method for detecting and eliminating such bias may not be trustworthy if statistical significance was required for the publication of most of the study estimates. We, therefore, performed worst-case scenario simulations with the mean sample size (Seiler et al., 2013) and error of measurement (3.1%) for the HIIT group in the $\dot{V}$ O_2max_ studies to determine the published mean effect for different true mean effects when only significant effects are published. A true effect of 10% would result in negligible upward publication bias, and substantial bias becomes evident only for true effects of 5% (published mean effect 6%) [HPW and WGH (Wiesinger et al., 2024a)]. We can, therefore, be very confident that the meta-analyzed mean effects of ∼10% for $\dot{V}$ O_2max_ do not suffer from publication bias, but the meta-analyzed mean effects for the other measures could be overestimated by ∼1–2% in the worst-case scenario.

## 5 Limitations and further research

What authors report in the included studies predefines what can be achieved in a meta-analysis. For these meta-analyses, the subject and study characteristics were poorly reported. We addressed some gaps by imputing a few missing values (Supplementary Tables S4–S9 and the HIIT spreadsheet), but insufficient descriptions of the HIIT and conventional training restricted our ability to include additional effect modifiers, which would have reduced heterogeneity. These deficiencies include information on supervised vs. non-supervised training, time of HIIT implementation within the training session, the period between the last training and post-test, net training and recovery time per session, mode and intensity of recovery periods, and environmental factors such as temperature or altitude. Measures of internal training loads, such as heart rate and time in zone, perceived exertion, blood lactate, oxygen consumption during the interval and recovery period, or training impulse, were also under-reported (Borresen and Lambert, 2009).

The inadequate reporting of subject characteristics was particularly critical for the meta-analysis of the effects of HIIT on $\dot{V}$ O_2max_. Four (Breil et al., 2010; Hanstock et al., 2020; Laursen et al., 2002; Stevens et al., 2015) of the twenty-two studies included in this analysis reported only absolute $\dot{V}$ O_2max_, while six (Sandbakk et al., 2013; Smith et al., 2003; Salazar-Martinez et al., 2018; Wells et al., 2014; Liu et al., 2021; Yang et al., 2017) focused solely on changes in relative $\dot{V}$ O_2max_. The absence of data on changes in body mass or body composition restricted our ability to perform accurate metric conversions between these two measures. To avoid excluding studies, we combined the findings from the 4 studies reporting absolute $\dot{V}$ O_2max_ with those from the 18 studies reporting relative $\dot{V}$ O_2max_. We prioritized changes in relative $\dot{V}$ O_2max_ because this metric accounts for changes in body mass and should more accurately predict changes in most types of athletic endurance performance. For the 12 studies reporting both metrics, a simple averaging of the changes showed a 0.4% greater improvement in relative $\dot{V}$ O_2max_ than in absolute $\dot{V}$ O_2max_. Owing to this negligible difference and the fact that the paper is already very extensive, we chose not to conduct a separate meta-analysis focused on absolute values.

For quantifying the mean effect of training, we recommend including values for changes in speed or power as these are practically the most relevant metrics and avoid additional uncertainties in meta-analyses by converting disparate measure effects to these units. Furthermore, we echo the call by previous meta-analysts (Weston et al., 2014; Wiesinger et al., 2015), encouraging authors to report exact inferential statistics (preferably means and SDs of change scores in experimental and control groups but definitely not *p*-value inequalities or their equivalent “significant” and “non-significant”).

Furthermore, team or racket sports athletes should consider using types of HIIT and test protocols that better reflect their competition demands. We adhered to the principle of training and testing specificity (Wiesinger et al., 2021; Buchheit, 2012; Hawley, 2008) by excluding studies of an apparent discrepancy between HIIT, testing methods, and the sport under investigation (Seo et al., 2019; Ojeda-Aravena et al., 2021; Herrera-Valenzuela et al., 2021; Thom et al., 2020; Sarkar et al., 2021). Nonetheless, most HIIT and testing methods in the studies included in our meta-analyses do not reflect the typical requirements of these sports, which involve changes in direction and maximal acceleration and deceleration (Meylan and Malatesta, 2009; Sporis et al., 2010). Except for one study using small-sided soccer games such as HIIT (Akdoğan et al., 2021) and a few shuttle sprint HIIT sessions (Iaia et al., 2009; Chtara et al., 2017), all anaerobic HIIT types consisted of linear sprints (Helgerud et al., 2001; Soares-Caldeira et al., 2014; Thomassen et al., 2010; Venturelli et al., 2008; Hermassi et al., 2018; Selmi et al., 2018; Dupont et al., 2004). Similarly, all tests measuring sprint speed/power and repeated-sprint ability were conducted linearly, even though validated sport-specific tests are available [e.g., Copenhagen Soccer Test (Sporis et al., 2010) and Bangsbo intermittent field tests (Samozino et al., 2016)]. None of these studies provided data on the highest acceleration, speed, or metrics related to the horizontal force–velocity profile (Samozino et al., 2016). Regarding cycle ergometer tests, information on maximal power was limited to Wingate tests.

To contextualize the principle of training and testing specificity further, we acknowledge potential differences between laboratory and field tests. Athletes participating in study settings are subject to expectation effects, while in actual competition, they may experience reduced or absent expectation effects due to high motivation to perform their best (Clark et al., 2000; Beedie and Foad, 2009). Therefore, the effects of HIIT observed in these meta-analyses may be larger than those occurring in competition performance (Vandenbogaerde et al., 2012), which unfortunately have not yet been studied. For future studies, we recommend documentation of competition performance before and after HIIT, in addition to sport-specific test performance.

Another limitation is the small number of study estimates, resulting in inadequate precision for training effects and the modifiers of the effects. We meta-analyzed the effect of HIIT on exercise economy, but for this performance predictor, we included only one effect modifier. The aerobic/anaerobic threshold was another predictor of performance with a small number of study estimates. For this measure, we addressed the limited data by including four studies (Sandbakk et al., 2013; Smith et al., 2003; Sandbakk et al., 2011; Sheykhlouvand et al., 2018a) wherein authors reported fractional utilization rather than speed/power. Although the mean effect of fractional utilization on changes in $\dot{V}$ O_2_ could theoretically be biased by changes in exercise economy, it is noteworthy that the type of HIIT used in most of these studies (Sandbakk et al., 2013; Smith et al., 2003; Sandbakk et al., 2011) had a negligible effect on exercise economy [see Wiesinger et al. (2024b)]. Owing to the limited amount of data, we opted to analyze the aerobic/anaerobic threshold as a practical measure (speed/power) rather than as a percent of $\dot{V}$ O_2max_.

For several reasons, we limited our analysis of errors of measurement to mean values: this article is focused on mean effects and between-study heterogeneity and is already arguably too extensive; a meta-analysis of the SD representing individual responses derived from the errors of measurement would require a different type of mixed model; and for some measures, there would simply not be enough data since for these measures more than half the errors of measurement could not be estimated from the data in the published studies (Table 1). The meta-analysis of individual responses is as important as the meta-analysis of mean effects, but more authors will have to provide standard deviations of change scores or other equivalent inferential information in experimental and control groups to allow such meta-analyses in the future.

Finally, four studies that meet our inclusion criteria have been published since the date of our literature search and subsequent analyses: two involving classical endurance athletes [cyclists (Christensen et al., 2024) and runners (Possamai et al., 2024)] and two involving other athletes [ice-hockey (Jeppesen et al., 2022) and field-hockey players (Taylor and Jakeman, 2022)]. The outcomes of these studies are consistent with the mean effects observed in our meta-analyses. These additional studies, therefore, provide further support for our findings and would not alter the overall conclusions.

## 6 Conclusion

For top athletes, HIIT outperforms conventional training in most study settings for most performance measures. HIIT was generally more effective when added to conventional training during the competition phase. The modifying effect of the type of HIIT was clear for $\dot{V}$ O_2max_ such that the strategy for improving athletes who are deficient in $\dot{V}$ O_2max_ is the most aerobic type of HIIT. Identifying the HIIT tools for improving deficiencies in other measures of performance will require more research, as will quantifying adequately the following effects: HIIT on exercise economy; HIIT with female athletes; and the modifying effects of the duration of intervention and the phase of training. Future studies could also focus on sport-specific HIIT in swimming and other relevant sports.

## Data Availability

The original contributions presented in the study are included in the article/Supplementary Material; further inquiries can be directed to hans-peter.wiesinger@pmu.ac.at.
