# Supplementary material for: Meta-analyses of the effects of high-intensity interval training in elite athletes—part I: mean effects on various performance measures
Source: Front Physiol. 2025 Jan 3;15:1486526. doi: 10.3389/fphys.2024.1486526 (PMC11739151; doi:10.3389/fphys.2024.1486526)
Supplement: Supplementary file 1 [file Table1.docx]

**Title**

Meta-analyses of the effects of high-intensity interval training in elite athletes – Part I: mean effects on various performance measures

**Supplementary appendix**

**This appendix formed part of the original submission**

**Running title**

Effects of HIIT on various performance measures

**Authors**

Hans-Peter Wiesinger^a,b,c,†,*^, Thomas Leonard Stöggl^a,d,†^, Nils Haller^a,e^, Julia Blumkaitis^a^, Tilmann Strepp^a^, Francesca Kilzer^a^, Anna Schmuttermair^d^, Will G. Hopkins^f^

^†^shared first author

^a^ Department of Sport and Exercise Science, Paris Lodron University Salzburg, Salzburg 5020, Austria.

^b^ Institute of Nursing Science and Practice, Center for Public Health and Healthcare Research, Paracelsus Medical University, 5020 Salzburg, Austria

^c^ Institute of General Practice, Family Medicine and Preventive Medicine, Center for Public Health and Healthcare Research, Paracelsus Medical University, 5020 Salzburg, Austria

^d^ Red Bull Athlete Performance Center, 5303 Thalgau, Austria

^e^ Department of Sports Medicine, Rehabilitation and Disease Prevention, Johannes Gutenberg University, Mainz 55122, Germany

^f^ Internet Society for Sport Science, Auckland, New Zealand

*Corresponding author:

Hans-Peter Wiesinger (ORCID: 0000-0001-8526-2832)

Institute of General Practice, Family Medicine and Preventive Medicine, Center for Public Health and Healthcare Research, Paracelsus Medical University

Strubergasse 21

5020 Salzburg

Austria

hans-peter.wiesinger@pmu.ac.at

Mobil: +43 (0) 699 19116380

**List of Tables**

[Table 1. Quality assessment criteria, adapted from Galna et al. [1] 4](#_Toc173222021)

[Table 2. Quality assessment scores 4](#_Toc173222022)

[Table 3. Study and subject characteristics included in the meta-analysis of effects of HIIT on **sprint** **speed/power** output for male non-endurance athletes. 5](#_Toc173222023)

[Table 4. Study and subject characteristics included in the meta-analysis of effects of HIIT on r**epeated-sprint ability** (mean sprint speed/power) for non-endurance trained athletes. 7](#_Toc173222024)

[Table 5. Study and subject characteristics included in the meta-analysis of effects of HIIT on **time-trial speed/power** output for endurance-trained athletes. 8](#_Toc173222025)

[Table 6. Study and subject characteristics included and excluded in the meta-analysis of effects of HIIT on **peak speed/power** in an incremental test for male athletes. 10](#_Toc173222026)

[Table 7. Study and subject characteristics included in the meta-analysis of effects of HIIT on **aerobic/anaerobic threshold** for non-endurance trained athletes. 12](#_Toc173222027)

[Table 8. Study and subject characteristics included and excluded in the meta-analysis of effects of HIIT on **V̇O_2max_**. 14](#_Toc173222028)

[Table 9. Study and subject characteristics included in the meta-analysis of effects of HIIT on **exercise economy** for endurance-trained athletes. 17](#_Toc173222029)

[Table 10: Summary of Tables 4 to 10 in the original manuscript, including predicted effects, moderator effects, and heterogeneity SDs in the meta-analyses of each performce measure. Error of measurement and individual-responses SDs are also included. The data are effect qualitative magnitude, probability of the magnitude, and the observed quantitative magnitude. 18](#_Toc173222030)

Search string

The search string used in the electronic bibliographic databases PubMed, Scopus, SPORTDiscus, and Web of Science, was conducted in the title, abstract and keywords, combined a population of highly endurance-trained individuals with different aerobic, anaerobic or neuromuscular performance-related outcomes” athlete*” OR “elite” OR “welltrained” OR “well trained” OR “international level” OR “trained” OR “first league” OR “premier league” OR “division 1” OR “national team” AND “high intensity interval training” OR “high intensity intermittent training” OR “high intensity training” OR “HIIT” OR “HIIT” OR “sprint interval training” OR “repeated sprint training” OR “SIT” OR “repeated sprint exercise*” OR “RSE” OR “RST” OR “RSA” OR “multiple sprint” OR “repeated-sprint sequence*” OR “high intensity endurance training” OR “speed endurance training” OR “speed endurance production” OR “speed endurance maintenance” OR “aerob* exercise” OR “aerob* high intensity” OR “Tabata” OR “all-out” OR “wingate” OR “anaerob* exercise” OR “anaerob* high intensity” and filtered for „humans“, „journal article“, „peer reviewed“, „English“ and „German“ if possible, with the search settings of the databases on the World Wide Web.

| Table 1. Quality assessment criteria, adapted from Galna et al. [1] | | |
| --- | --- | --- |
| **Criterion** | **Question** | **Categories** |
| 1 | Research aims or questions stated clearly | 1-yes; 0.5 yes; lacking  detail or clarity; 0 -no |
| 2 | Participants detailed | number, age, sex, height, body mass |
| 3 | Recruitment and sampling methods described | 1-yes; 0.5 yes; lacking  detail or clarity; 0 -no |
| 4 | Inclusion and exclusion criteria detailed | 1-yes; 0.5 yes; lacking  detail or clarity; 0 -no |
| 5 | Controlled co-variates | age, sex, baseline values |
| 6 | Key outcome variables clearly described | 1-yes; 0.5 - only some defined; 0.5-yes, lacking detail or clarity; 0-no |
| 7 | Adequate methodology able to repeat study | participants sampling, equipment, procedure, data processing, statistical analysis |
| 8 | Methodology able to answer research question | participants sampling, equipment, procedure, data processing, statistical analysis |
| 9 | Reliability of the methodology stated | 1-yes, 0.5 yes, lacking  detail or clarity; 0 -no |
| 10 | Internal validity of the methodology stated | 1- yes, 0 - no |
| 11 | Research questions answered adequately in the discussion | 1- yes, 0 - no |
| 12 | Key findings supported by the results | 1- yes, 0 - no |
| 13 | Key finding interpreted in a logical manner which is supported by references | 1- yes, 0 - no |

| Table 2. Quality assessment scores | | | | | | | | | | | | | | | |
| --- | --- | --- | --- | --- | --- | --- | --- | --- | --- | --- | --- | --- | --- | --- | --- |
| **Source for endurance athletes** | **Criterion** | | | | | | | | | | | | | | **Total** |
|  | **1** | **2** | **3** | **4** | **5** | **6** | **7** | **8** | **9** | **10** | **11** | **12** | **13** | **(0-13)** | |
| Clark et al. [2] | 0.5 | 0.7 | 0.5 | 0 | 0 | 0.5 | 0.9 | 0.9 | 0 | 1 | 1 | 1 | 1 | 8.0 | |
| Hanstock et al. [3] | 1 | 0.5 | 0.5 | 0 | 0.5 | 0.5 | 1 | 1 | 0 | 1 | 1 | 1 | 1 | 8.8 | |
| Johansen et al. [4] | 1 | 1 | 0.5 | 0.5 | 0 | 1 | 1 | 1 | 0 | 0 | 1 | 1 | 1 | 9.0 | |
| Laursen et al. [5] | 1 | 1 | 0.5 | 0.5 | 0 | 1 | 1 | 1 | 0 | 0 | 1 | 1 | 1 | 10.5 | |
| Menz et al. [6] | 1 | 1 | 0.5 | 0.5 | 0.5 | 1 | 1 | 1 | 0 | 1 | 1 | 1 | 1 | 10.5 | |
| Rønnestad et al. [7] | 1 | 0.7 | 0.5 | 0.5 | 0 | 1 | 0.9 | 0.9 | 0.5 | 1 | 1 | 1 | 1 | 10.0 | |
| Salazar-Martínez et al. [8] | 1 | 1 | 0.5 | 0.5 | 0 | 1 | 1 | 1 | 0 | 1 | 1 | 1 | 1 | 10.0 | |
| Sandbakk et al. [9] | 1 | 1 | 0.5 | 1 | 0 | 1 | 1 | 1 | 0 | 1 | 1 | 1 | 1 | 10.5 | |
| Sandbakk et al. [10] | 1 | 0.7 | 0.5 | 0 | 0.5 | 1 | 0.9 | 0.9 | 0.5 | 1 | 1 | 1 | 1 | 10.0 | |
| Skovereng et al. [11] | 1 | 0.7 | 0.5 | 0 | 0.5 | 1 | 0.9 | 0.9 | 0.5 | 1 | 1 | 1 | 1 | 10.0 | |
| Smith et al. [12] | 1 | 0.5 | 0.5 | 0 | 0 | 1 | 0.8 | 0.9 | 0 | 1 | 1 | 1 | 1 | 8.7 | |
| Stenqvist et al. [13] | 0.5 | 1 | 0.5 | 1 | 0 | 1 | 1 | 0.9 | 0.5 | 0.5 | 1 | 1 | 1 | 10.4 | |
| Stepto et al. [14] | 0.5 | 1 | 0.5 | 0 | 0 | 1 | 0.9 | 0.9 | 0 | 1 | 1 | 1 | 1 | 8.8 | |
| Stevens et al. [15] | 1 | 0.8 | 0.5 | 0 | 0 | 1 | 0.8 | 0.9 | 0.5 | 1 | 1 | 1 | 1 | 9.5 | |
| Stöggl et al. [16] | 1 | 0.6 | 0.5 | 0.5 | 0 | 1 | 1 | 0.9 | 0.5 | 1 | 1 | 1 | 1 | 10.0 | |
| Sylta et al. [17] | 1 | 0.6 | 1 | 1 | 0.5 | 1 | 0.9 | 0.9 | 0 | 1 | 1 | 1 | 1 | 10.9 | |
| **Source for non-endurance athletes** | **Criterion** | | | | | | | | | | | | | **Total** | |
|  | **1** | **2** | **3** | **4** | **5** | **6** | **7** | **8** | **9** | **10** | **11** | **12** | **13** | **(0-13)** | |
| Akdoğan et al. [18] | 1 | 0.8 | 0.5 | 0.5 | 0 | 1 | 1 | 0.9 | 0 | 1 | 1 | 1 | 1 | 9.7 | |
| Breil et al. [19] | 1 | 1 | 0.5 | 0.5 | 0.5 | 1 | 0.9 | 0.9 | 0.5 | 1 | 1 | 1 | 1 | 10.8 | |
| Chtara et al. [20] | 1 | 0.7 | 0.5 | 0 | 0 | 1 | 0.9 | 0.9 | 1 | 1 | 1 | 1 | 1 | 10.0 | |
| Dupont et al. [21] | 1 | 0.7 | 0.5 | 0 | 0 | 1 | 0.8 | 0.7 | 0 | 0 | 1 | 1 | 1 | 6.7 | |
| Helgerud et al. [22] | 1 | 0.7 | 0.5 | 0.5 | 0 | 1 | 0.8 | 1 | 1 | 1 | 1 | 1 | 1 | 10.5 | |
| Hermassi et al. [23] | 0.5 | 1 | 0.5 | 0.5 | 0 | 1 | 1 | 0.9 | 1 | 1 | 1 | 1 | 1 | 10.4 | |
| Iaia et al. [24] | 1 | 1 | 1 | 1 | 0 | 1 | 1 | 0.9 | 0 | 1 | 1 | 1 | 1 | 10.9 | |
| Liu et al. [25] | 1 | 1 | 0.5 | 0.5 | 0 | 1 | 1 | 0.9 | 0 | 1 | 1 | 1 | 1 | 9.9 | |
| Purkhús et al. [26] | 1 | 1 | 0.5 | 0.5 | 0 | 1 | 0.6 | 0.9 | 0.5 | 0 | 1 | 1 | 1 | 9.0 | |
| Selmi et al. [27] | 1 | 0.5 | 0.5 | 0.5 | 0 | 1 | 0.8 | 0.8 | 0 | 1 | 1 | 1 | 1 | 9.1 | |
| Sheykhlouvand et al. [28] | 1 | 0.7 | 0.5 | 0.5 | 0 | 1 | 0.9 | 1 | 0.5 | 1 | 1 | 1 | 1 | 10.1 | |
| Sheykhlouvand et al. [29] | 1 | 0.7 | 0.5 | 0.5 | 0 | 1 | 0.9 | 1 | 0 | 1 | 1 | 1 | 1 | 9.6 | |
| Sheykhlouvand et al. [30] | 1 | 0.7 | 0.5 | 0.5 | 1 | 1 | 0.9 | 1 | 0 | 1 | 1 | 1 | 1 | 10.6 | |
| Soares-Caldeira et al. [31] | 1 | 0.8 | 0.5 | 1 | 0 | 1 | 0.8 | 0.9 | 0.5 | 1 | 1 | 1 | 1 | 10.5 | |
| Thomassen et al. [32] | 0.5 | 0.7 | 0.5 | 0 | 0 | 1 | 1 | 0.9 | 0.5 | 1 | 1 | 1 | 1 | 9.1 | |
| Venturelli et al. [33] | 1 | 1 | 1 | 1 | 0 | 1 | 1 | 0.9 | 0 | 1 | 1 | 1 | 1 | 10.9 | |
| Wells et al. [34] | 0.5 | 0.7 | 0.5 | 0.5 | 0 | 1 | 1 | 0.9 | 0 | 1 | 1 | 1 | 1 | 9.1 | |
| Yang et al. [35] | 0.5 | 1 | 0.5 | 0.5 | 0 | 1 | 0.9 | 0.9 | 0 | 1 | 1 | 1 | 1 | 9.3 | |

| Table 3. Study and subject characteristics included in the meta-analysis of effects of HIIT on sprint speed/power output for male non-endurance athletes. | | | | | | | | | | | |
| --- | --- | --- | --- | --- | --- | --- | --- | --- | --- | --- | --- |
| **Study** | **Year** | **Treatment abbreviation** | **Our**  **treatment** | **Type of HIIT^a^** | **Sample size** | **Phase of training^b^** | **Extra training^c^** | **Intervention duration (wk)** | **Pre-test time**  **(s)** | **Effect (%)**  **Mean; 90% CL** |  |
| Akdoğan et al. [18] | 2021 | CG | Control |  | 9 | 1 | 0 | 6 | 2 | -5.4; -7.3 to -3.6 |  |
|  |  | CG | Control |  | 9 | 1 | 0 | 6 | 4 | -0.4; -2.4 to 1.5 |  |
|  |  | CG | Control |  | 9 | 1 | 0 | 6 | 5 | 0.4; -1.5 to 2.4 |  |
|  |  | SSG | HIIT | 1 | 11 | 1 | 0 | 6 | 2 | -4.0; -5.6 to -2.3 |  |
|  |  | SER | HIIT | 4 | 10 | 1 | 0 | 6 | 2 | -6.2; -7.9 to -4.5 |  |
|  |  | SSG | HIIT | 1 | 11 | 1 | 0 | 6 | 4 | -0.5; -2.1 to 1.3 |  |
|  |  | SER | HIIT | 4 | 10 | 1 | 0 | 6 | 4 | -3.4; -5.1 to -1.6 |  |
|  |  | SSG | HIIT | 1 | 11 | 1 | 0 | 6 | 5 | 0.8; -0.9 to 2.5 |  |
|  |  | SER | HIIT | 4 | 10 | 1 | 0 | 6 | 5 | -0.2; -2.0 to 1.6 |  |
| Chtara et al. [20] | 2017 | CON single | Control |  | 10 | 0 | 0 | 6 | 5 | 1.3; -0.5 to 3.2 |  |
|  |  | CON best | Control |  | 10 | 0 | 0 | 6 | 6 | 0.0; -1.8 to 1.8 |  |
|  |  | RS single | HIIT | 5 | 12 | 0 | 0 | 6 | 5 | 1.5; -0.1 to 3.2 |  |
| Chtara et al. [20] | 2017 | RS best | HIIT | 5 | 12 | 0 | 0 | 6 | 6 | 1.8; 0.1 to 3.4 |  |
| Dupont et al. [21] | 2004 | Con | Control |  | 22 | 0 | 0 | 10 | 6 | 0.2; -1.0 to 1.3 |  |
|  |  | Exp | HIIT | 3.5 | 22 | 0 | 0 | 10 | 6 | 3.7; 2.0 to 5.5 |  |
| Helgerud et al. [22] | 2001 | CON | Control |  | 10 | 0 | 0 | 8 | 2 | 0.0; -1.8 to 1.8 |  |
|  |  | CON | Control |  | 10 | 0 | 0 | 8 | 6 | -0.2; -2.0 to 1.7 |  |
|  |  | HIIT | HIIT | 1 | 9 | 0 | 0 | 8 | 2 | 0.5; -1.4 to 2.5 |  |
|  |  | HIIT | HIIT | 1 | 9 | 0 | 0 | 8 | 6 | 0.4; -1.6 to 2.3 |  |
| Hermassi et al. [23] | 2018 | Control | Control |  | 15 | 0 | 0 | 7 | 6 | 0.7; -0.8 to 2.1 |  |
|  |  | Intervention | HIIT | 4 | 15 | 0 | 1 | 7 | 6 | 3.0; 1.5 to 4.4 |  |
| Iaia et al. [24] | 2015 | SEP | HIIT | 4 | 6 | 0 | 0 | 3 | 3 | 0.4; -1.7 to 2.5 |  |
|  |  | SEM | HIIT | 3 | 7 | 0 | 0 | 3 | 3 | 1.4; -0.2 to 3.0 |  |
|  |  | SEP | HIIT | 4 | 6 | 0 | 0 | 3 | 5 | -0.4; -1.8 to 1.1 |  |
|  |  | SEM | HIIT | 3 | 7 | 0 | 0 | 3 | 5 | 0.8; -0.9 to 2.5 |  |
|  |  | SEP | HIIT | 4 | 6 | 0 | 0 | 3 | 26 | 1.2; 0.7 to 1.7 |  |
|  |  | SEM | HIIT | 3 | 7 | 0 | 0 | 3 | 27 | 2.2; 0.1 to 4.3 |  |
| Selmi et al. [27] | 2018 | Con-G | Control |  | 15 | 0 | 0 | 6 | 8 | -0.8; -1.9 to 0.4 |  |
|  |  | RST-G | HIIT | 5 | 15 | 0 | 0 | 6 | 8 | 4.4; 2.7 to 6.2 |  |
| Soares-Caldeira et al. [31] | 2014 | NormT | Control |  | 7 | 1 | 0 | 4 | 7 | 0.1; -1.6 to 1.9 |  |
|  |  | AddT | HIIT | 5 | 6 | 1 | 1 | 4 | 7 | 1.4; 0.1 to 2.7 |  |
| Thomassen et al. [32] | 2010 | In | Control |  | 11 | 1 | 0 | 2 | 3 | 0.0; -1.7 to 1.7 |  |
|  |  | HI | HIIT | 2.5 | 7 | 1 | 0 | 2 | 3 | 0.0; -2.3 to 2.3 |  |
| Venturelli et al. [33] | 2008 | STG | HIIT | 6 | 7 | 0 | 0 | 12 | 4 | 2.5; 0.3 to 4.7 |  |
| **Excluded studies** | | | | | | | | | | | |
| Sheykhlouvand et al. [28] | 2016 | Gcon | Control |  | 7 | 1 | 0 | 3 | 5 | 4.7; -0.3 to 9.9 |  |
|  |  | Gcon | Control |  | 7 | 1 | 0 | 3 | 30 | 3.5; -3.2 to 10.7 |  |
|  |  | G1 | HIIT | 3 | 7 | 1 | 0 | 3 | 5 | 9.7; 3.5 to 16.3 |  |
|  |  | G2 | HIIT | 3 | 7 | 1 | 0 | 3 | 5 | 12.2; 4.3 to 20.7 |  |
|  |  | G1 | HIIT | 3 | 7 | 1 | 0 | 3 | 30 | 10.7; 3.5 to 18.4 |  |
|  |  | G2 | HIIT | 3 | 7 | 1 | 0 | 3 | 30 | 16.1; 8.6 to 24.2 |  |
| Sheykhlouvand et al. [29] | 2018 | Control | Control |  | 7 | 1 | 0 | 4 | 5 | 0.5; -5.4 to 6.8 |  |
|  |  | Control | Control |  | 8 | 1 | 00 | 4 | 30 | 0.3; -5.6 to 6.7 |  |
|  |  | SIT | HIIT | 5 | 8 | 1 | 0 | 4 | 5 | 9.5; 1.8 to 17.7 |  |
|  |  | SIT | HIIT | 5 | 8 | 1 | 0 | 4 | 30 | 3.8; -2.3 to 10.4 |  |
| Yang et al. [35] | 2017 | HIIT | HIIT | 1.5 | 7 | 1 | 0 | 4 | 49 | 3.8; 1.8 to 5.8 |  |
|  |  | HIIT | HIIT | 1.5 | 7 | 1 | 0 | 4 | 138 | 1.8; -0.2 to 3.8 |  |
|  |  | HIIT | HIIT | 1.5 | 7 | 1 | 0 | 4 | 296 | 3.6; 1.6 to 5.7 |  |
| CI, confidence intervals.  Excluded studies: none.  ^a^ Range of 1 (aerobic traditional long intervals) to 6 (anaerobic sprint intervals), as presented in Figure 1 in Stöggl et al. [38]  ^b^ 0, during the competition phase; 1, outside the competition phase; 0.5, mixture of competition and pre- or post-competition phase. Missing values were imputed to the mean of eligible study estimates (shown in parentheses).  ^c^ 0, HIIT partly or entirely replaced regular training; 1, HIIT was added to regular training. | | | | | | | | | | | |

| Table 4. Study and subject characteristics included in the meta-analysis of effects of HIIT on repeated-sprint ability (mean sprint speed/power) for non-endurance trained athletes. | | | | | | | | | | | | |
| --- | --- | --- | --- | --- | --- | --- | --- | --- | --- | --- | --- | --- |
| **Study** | **Year** | **Treatment**  **abbreviation** | **Our treatment** | **Type of HIIT^a^** | **Sample size** | **Maleness** | **Phase of training^b^** | **Extra training^c^** | **Intervention duration (wk)** | **Pre-test decrement (%)** | **Effect (%)**  **Mean; 90% CL** |  |
| Akdoğan et al. [18] | 2021 | CG | Control |  | 9 | 1 | 1 | 0 | 6 | 5.5 | 0.2; -0.9 to 1.4 |  |
|  |  | SSG | HIIT | 1 | 11 | 1 | 1 | 0 | 6 | 3.2 | 0.6; -0.4 to 1.7 |  |
|  |  | SER | HIIT | 4 | 10 | 1 | 1 | 0 | 6 | 3.4 | -0.4; -1.5 to 0.7 |  |
| Chtara et al. [20] | 2017 | CON | Control |  | 10 | 1 | 0 | 0 | 6 | 3.5 | 1.1; -0.1 to 2.3 |  |
|  |  | RS | HIIT | 5 | 12 | 1 | 0 | 0 | 6 | 3.5 | 1.7; 0.7 to 2.8 |  |
| Hermassi et al. [23] | 2018 | Control | Control |  | 15 | 1 | 0 | 0 | 7 | 3.8 | -0.3; -1.2 to 0.7 |  |
|  |  | Intervention | HIIT | 4 | 15 | 1 | 0 | 1 | 7 | 3.7 | 2.7; 1.8 to 3.7 |  |
| Iaia et al. [24] | 2015 | SEP | HIIT | 4 | 6 | 1 | 0 | 0 | 3 | 5.0 | 2.5; 1.7 to 3.4 |  |
|  |  | SEM | HIIT | 3 | 7 | 1 | 0 | 0 | 3 | 4.1 | -1.0; -2.0 to 0.0 |  |
| Purkhús et al. [26] | 2016 | CON | Control |  | 12 | 0 | 0 | 0 | 8 | 5.5 | -0.2; -2.0 to 1.7 |  |
|  |  | HIIT | HIIT | 4 | 13 | 0 | 0 | 0 | 8 | 7.0 | 4.4; 2.0 to 6.9 |  |
| Selmi et al. [27] | 2018 | Con-G | Control |  | 15 | 1 | 0 | 0 | 6 | 6.3 | -1.0; -2.1 to 0.2 |  |
|  |  | RST-G | HIIT | 5 | 15 | 1 | 0 | 0 | 6 | 6.0 | 5.5; 3.5 to 7.6 |  |
| Soares-Caldeira et al. [31] | 2014 | NormT | Control |  | 7 | 1 | 1 | 0 | 4 | 7.8 | 2.9; 1.9 to 3.9 |  |
|  |  | AddT | HIIT | 5 | 6 | 1 | 1 | 0 | 4 | 6.3 | 2.6; 1.6 to 3.5 |  |
| Thomassen et al. [32] | 2010 | In | Control |  | 11 | 1 | 1 | 0 | 2 | 6.1 | -2.1; -3.0 to -1.2 |  |
|  |  | HI | HIIT | 3 | 7 | 1 | 1 | 0 | 2 | 5.2 | 1.9; 0.4 to 3.5 |  |
| CI, confidence intervals.  Excluded studies: none.  ^a^ Range of 1 (aerobic traditional long intervals) to 6 (anaerobic sprint intervals), as presented in Figure 1 in Stöggl et al. [38]  ^b^ 0, during the competition phase; 1, outside the competition phase; 0.5, mixture of competition and pre- or post-competition phase. Missing values were imputed to the mean of eligible study estimates (shown in parentheses).  ^c^ 0, HIIT partly or entirely replaced regular training; 1, HIIT was added to regular training. | | | | | | | | | | | | |

| Table 5. Study and subject characteristics included in the meta-analysis of effects of HIIT on **time-trial speed/power** output for endurance-trained athletes. | | | | | | | | | | | |
| --- | --- | --- | --- | --- | --- | --- | --- | --- | --- | --- | --- |
| **Study** | **Year** | **Treatment abbreviation** | **Our treatment** | **Type of HIIT^a^** | **Sample size** | **Maleness^b^** | **Extra training^c^** | **Intervention duration (wk)** | **Pre-test time (min)** | **Effect (%)**  **Mean; 90% CL** |  |
| Clark et al. [2] | 2014 | Control | Control |  | 9 | 1 | 0 | 1 | 38 | -3.1; -5.9 to -0.3 |  |
|  |  | Short HIIT | HIIT | 5 | 9 | 1 | 0 | 1 | 38 | 4.3; 2.3 to 6.3 |  |
|  |  | Long HIIT | HIIT | 4 | 10 | 1 | 0 | 1 | 39 | 6.9; 4.5 to 9.3 |  |
| Laursen et al. [5] | 2002 | Control | Control |  | 11 | 1 | 0 | 4 | 57 | -2.1; -4.3 to 0.2 |  |
|  |  | G1 | HIIT | 1 | 8 | 1 | 1 | 4 | 57 | 11.5; 6.6 to 16.6 |  |
|  |  | G2 | HIIT | 1 | 9 | 1 | 1 | 4 | 58 | 12.2; 8.8 to 15.8 |  |
|  |  | G3 | HIIT | 4 | 10 | 1 | 1 | 4 | 57 | 9.5; 4.7 to 14.4 |  |
| Salazar-Martínez et al. [8] | 2018 | Con | Control |  | 8 | 1 | 0 | 3 | 1 | 1.0; -3.4 to 5.6 |  |
|  |  | HIIT | HIIT | 1 | 8 | 1 | 0 | 3 | 1 | 2.2; 0.3 to 4.2 |  |
| Skovereng et al. [11] | 2018 | HIIT | HIIT | 1 | 63 | 1 | 1 | 12 | 40 | 6.7; 4.8 to 8.7 |  |
| Smith et al. [12] | 2003 | CON | Control |  | 9 | (1) | 0 | 4 | 10 | 0.1; -2.2 to 2.4 |  |
|  |  | CON | Control |  | 9 | (1) | 0 | 4 | 18 | -0.9; -3.1 to 1.3 |  |
|  |  | 60%Tmax | HIIT | 1 | 9 | (1) | 0 | 4 | 11 | 2.8; 1.8 to 3.9 |  |
|  |  | 70%Tmax | HIIT | 1 | 9 | (1) | 0 | 4 | 10 | 1.0; -0.2 to 2.3 |  |
|  |  | 60%Tmax | HIIT | 1 | 9 | (1) | 0 | 4 | 19 | 2.3; 0.1 to 4.7 |  |
|  |  | 70%Tmax | HIIT | 1 | 9 | (1) | 0 | 4 | 18 | 0.3; -1.6 to 2.3 |  |
| Stepto et al. [14] | 1999 | HIIT | HIIT | 4 | 11 | 1 | 0 | 3 | 57 | 3.2; 1.0 to 5.4 |  |
| Stevens et al. [15] | 2015 | EBTAlone | Control |  | 8 | (1) | 0 | 4 | 7 | 1.2; 0.5 to 1.8 |  |
|  |  | EBTSIT | HIIT | 4 | 8 | (1) | 0 | 4 | 7 | 2.9; 2.0 to 3.8 |  |
| Sylta et al. [17] | 2016 | INC | HIIT | 1 | 23 | 1 | 0 | 12 | 40 | 8.2; 5.4 to 11.0 |  |
|  |  | DEC | HIIT | 1 | 20 | 1 | 0 | 12 | 40 | 6.8; 4.2 to 9.5 |  |
|  |  | MIX | HIIT | 1 | 20 | 1 | 0 | 12 | 40 | 3.5; 1.7 to 5.3 |  |
|  |  | INC | HIIT | 1 | 23 | 1 | 0 | 12 | 0.5 | 1.2; -0.4 to 2.7 |  |
|  |  | DEC | HIIT | 1 | 20 | 1 | 0 | 12 | 0.5 | 2.4; 0.8 to 4.1 |  |
|  |  | MIX | HIIT | 1 | 20 | 1 | 0 | 12 | 0.5 | 2.2; 0.4 to 4.0 |  |
| **Excluded studies** | | | | | | | | | | |  |
| Sandbakk et al. [9] | 2011 | CON | Control |  | 8 | 0.33 | 0 | 8 | 4 | -0.9; -2.2 to 0.4 |  |
|  |  | IG | HIIT | 1 | 7 | 0.33 | 0 | 8 | 4 | 4.7; 2.4 to 7.1 |  |
| Sandbakk et al. [10] | 2013 | CON | Control |  | 7 | 0.57 | 1 | 8 | 32 | 1.0; -0.3 to 2.4 |  |
|  |  | CON | Control |  | 7 | 0.57 | 1 | 8 | 35 | 1.0; -1.5 to 3.5 |  |
|  |  | SIG | HIIT | 1 | 7 | 0.57 | 1 | 8 | 33 | 1.6; -0.1 to 3.2 |  |
|  |  | LIG | HIIT | 1 | 7 | 0.57 | 1 | 8 | 33 | 4.7; 2.8 to 6.7 |  |
| Sandbakk et al. [10] | 2013 | SIG | HIIT | 1 | 7 | 0.57 | 1 | 8 | 37 | 0.9; -1.5 to 3.4 |  |
|  |  | LIG | HIIT | 1 | 7 | 0.57 | 1 | 8 | 37 | 6.8; 3.8 to 10.0 |  |
| Stevens et al. [15] | 2015 | EBTAlone | Control |  | 8 | (1) | 0 | 4 | 1 | 0.4; -1.4 to 2.2 |  |
|  |  | EBTSIT | HIIT | 4 | 8 | (1) | 0 | 4 | 1 | 4.4; 1.1 to 7.8 |  |
| CI, confidence intervals.  Excluded studies: none.  ^a^ Range of 1 (aerobic traditional long intervals) to 6 (anaerobic sprint intervals), as presented in Figure 1 in Stöggl et al. [38]  ^b^ Proportion of males; missing values were imputed to 1 (shown in parentheses).  ^c^ 0, during the competition phase; 1, outside the competition phase; 0.5, mixture of competition and pre- or post-competition phase. Missing values were imputed to the mean of eligible study estimates (shown in parentheses).  ^c^ 0, HIIT partly or entirely replaced regular training; 1, HIIT was added to regular training. | | | | | | | | | | | |

| Table 6. Study and subject characteristics included and excluded in the meta-analysis of effects of HIIT on peak speed/power in an incremental test for male athletes. | | | | | | | | | | | | | | |  |
| --- | --- | --- | --- | --- | --- | --- | --- | --- | --- | --- | --- | --- | --- | --- | --- |
| **Study** | **Year** | **Type of athlete** | **Treatment abbreviation** | **Our treatment** | **Type of test** | **Protocol** | **Type of HIIT^a^** | **Sample size** | **Phase of training^b^** | **Extra training^c^** | **Intervention duration (wk)** | **Adjusted test duration (min)** | **Effect (%)**  **Mean; 90% CL** |  |  |
| Akdoğan et al.[18] | 2021 | Other | CG | Control | Yoyo | Yoyo IR1 |  | 9 | 1 | 0 | 6 | 8 | 0.4; -0.7 to 1.5 |  |  |
|  |  | Other | CG | Control | Yoyo | Yoyo IR2 |  | 9 | 1 | 0 | 6 | 4 | 0.1; -1.2 to 1.4 |  |  |
|  |  | Other | SSG | HIIT | Yoyo | Yoyo IR1 | 1 | 11 | 1 | 0 | 6 | 11 | 2.6; 1.4 to 3.7 |  |  |
|  |  | Other | SER | HIIT | Yoyo | Yoyo IR1 | 4 | 10 | 1 | 0 | 6 | 11 | 3.2; 2.2 to 4.3 |  |  |
|  |  | Other | SSG | HIIT | Yoyo | Yoyo IR2 | 1 | 11 | 1 | 0 | 6 | 4 | 2.1; 1.0 to 3.3 |  |  |
|  |  | Other | SER | HIIT | Yoyo | Yoyo IR2 | 4 | 10 | 1 | 0 | 6 | 4 | 2.3; 1.1 to 3.5 |  |  |
| Clark et al. [2] | 2014 | Endurance | Control | Control | Incr. | CycleIncr. |  | 9 | 0 | 0 | 1 | 18 | -1.8; -3.5 to 0.0 |  |  |
|  |  | Endurance | Short HIIT | HIIT | Incr. | CycleIncr. | 5 | 9 | 0 | 0 | 1 | 19 | 7.6; 3.6 to 11.7 |  |  |
|  |  | Endurance | Long HIIT | HIIT | Incr. | CycleIncr. | 4 | 10 | 0 | 0 | 1 | 19 | 3.6; 2.9 to 4.2 |  |  |
| Iaia et al. [24] | 2015 | Other | SEP | HIIT | Yoyo | Yoyo IR2 | 4 | 6 | 0 | 0 | 3 | 7 | 0.8; 0.4 to 1.2 |  |  |
|  |  | Other | SEM | HIIT | Yoyo | Yoyo IR2 | 3 | 7 | 0 | 0 | 3 | 7 | 0.3; -0.1 to 0.7 |  |  |
| Laursen et al. [5] | 2002 | Endurance | Control | Control | Incr. | CycleIncr. |  | 11 | 1 | 0 | 4 | 7 | -0.9; -1.8 to -0.1 |  |  |
|  |  | Endurance | G1 | HIIT | Incr. | CycleIncr. | 1 | 8 | 1 | 1 | 4 | 7 | 4.8; 2.8 to 6.8 |  |  |
|  |  | Endurance | G2 | HIIT | Incr. | CycleIncr. | 1 | 9 | 1 | 1 | 4 | 7 | 6.0; 4.3 to 7.8 |  |  |
|  |  | Endurance | G3 | HIIT | Incr. | CycleIncr. | 4 | 10 | 1 | 1 | 4 | 7 | 3.1; 1.3 to 4.8 |  |  |
| Liu et al. [25] | 2021 | Other | CON male | Control | Yoyo | Yoyo IR2 |  | 8 | 0 | 0 | 8 | 7 | 0.4; -0.6 to 1.4 |  |  |
|  |  | Other | SIT male | HIIT | Yoyo | Yoyo IR2 | 4 | 8 | 0 | 0 | 8 | 8 | 1.1; 0.1 to 2.1 |  |  |
| Rønnestad et al. [7] | 2019 | Endurance | Exp | HIIT | Incr. | CycleIncr. | 3 | 9 | 0 | 1 | 0.9 | 7 | 5.0; 2.4 to 7.6 |  |  |
| Skovereng et al. [11] | 2018 | Endurance | HIIT | HIIT | Incr. | CycleIncr. | 1 | 63 | 1 | 1 | 12 | 5 | 3.2; 1.6 to 4.9 |  |  |
| Soares-Caldeira et al. [31] | 2014 | Other | NormT | Control | Yoyo | Yoyo IR1 |  | 7 | 1 | 0 | 4 | 10 | 2.9; 1.6 to 4.3 |  |  |
|  |  | Other | AddT | HIIT | Yoyo | Yoyo IR1 | 5 | 6 | 1 | 1 | 4 | 10 | 3.7; 2.0 to 5.4 |  |  |
| Stepto et al. [14] | 1999 | Endurance | HIIT | HIIT | Incr. | CycleIncr. | 4 | 11 | 0 | 0 | 3 | 12 | 1.1; 0.5 to 1.8 |  |  |
| Stöggl et al. [16] | 2014 | Endurance | HVTcyc | Control | Incr. | CycleIncr. |  | 3 | 0.5 | 0 | 9 | 3 | 2.5; 0.5 to 4.5 |  |  |
|  |  | Endurance | HVTrun | Control | Incr. | RunIncr. |  | 5 | 0.5 | 0 | 9 | 4 | 5.6; 0.8 to 10.7 |  |  |
|  |  | Endurance | HIIT | HIIT | Incr. | CycleIncr. | 1 | 9 | 0.5 | 0 | 9 | 4 | 3.9; 1.8 to 6.1 |  |  |
|  |  | Endurance | POLcyc | HIIT | Incr. | CycleIncr. | 1 | 6 | 0.5 | 0 | 9 | 3 | 5.7; 2.8 to 8.6 |  |  |
| Sylta et al. [17] | 2016 | Endurance | INC | HIIT | Incr. | CycleIncr. | 1 | 23 | 1 | 0 | 12 | 6 | 4.6; 2.3 to 6.8 |  |  |
|  |  | Endurance | DEC | HIIT | Incr. | CycleIncr. | 1 | 20 | 1 | 0 | 12 | 6 | 5.3; 3.6 to 7.1 |  |  |
|  |  | Endurance | MIX | HIIT | Incr. | CycleIncr. | 1 | 20 | 1 | 0 | 12 | 6 | 6.1; 3.5 to 8.7 |  |  |
| Thomassen et al. [32] | 2010 | Other | In | Control | Yoyo | Yoyo IR2 |  | 11 | 1 | 0 | 2 | 6 | -0.4; -0.5 to -0.2 |  |  |
|  |  | Other | HI | HIIT | Yoyo | Yoyo IR2 | 3 | 7 | 1 | 0 | 2 | 7 | 0.5; -0.2 to 1.2 |  |  |
| Wells et al. [34] | 2014 | Other | CON | Control | Incr. | RunIncr. |  | 8 | 0 | 0 | 6 | 8 | -2.1; -3.2 to -1.0 |  |  |
| Wells et al. [34] | 2014 | Other | CON MART | Control | Incr. | RunIncr. |  | 8 | 0 | 0 | 6 | 2 | 1.3; -2.4 to 5.3 |  |  |
|  |  | Other | TRA | HIIT | Incr. | RunIncr. | 4 | 8 | 0 | 0 | 6 | 8 | -0.5; -1.5 to 0.5 |  |  |
|  |  | Other | TRA MART | HIIT | Incr. | RunIncr. | 4 | 8 | 0 | 0 | 6 | 2 | 9.8; 3.2 to 16.9 |  |  |
| **Excluded studies** | | | | | | | | | | | | | | | |
| Hermassi et al. [23] | 2018 | Other | Control | Control | Yoyo | Yoyo IR2 |  | 15 | 0 | 0 | 7 | 4 | -2.9; -3.6 to -2.2 |  |  |
|  |  | Other | Intervention | HIIT | Yoyo | Yoyo IR2 | 4 | 15 | 0 | 1 | 7 | 4 | 25.9; 25.0 to 26.8 |  |  |
| Sarkar et al. [37] | 2021 | Other | Control | Control | Incr. | CycleIncr. |  | 20 |  | 0 | 8 |  | -0.9; -2.1 to 0.3 |  |  |
|  |  | Other | HIIT | HIIT | Incr. | CycleIncr. | n.i. | 20 |  | 0 | 8 |  | 11.6; 6.2 to 17.2 |  |  |
| CI, confidence intervals; Incr., incremental test; Yoyo IR1, Yoyo intermittent recovery test level 1; Yoyo IR2, Yoyo intermittent recovery test level 2.  Excluded studies: The outcome measure in Hermassi et al. [23] was an extreme outlier, Sarkar et al. [37], did not have clear information about testing.  ^a^ Range of 1 (aerobic traditional long intervals) to 6 (anaerobic sprint intervals), as presented in Figure 1 in Stöggl et al. [38]  ^b^ 0, during the competition phase; 1, outside the competition phase; 0.5, mixture of competition and pre- or post-competition phase. Missing values were imputed to the mean of eligible study estimates (shown in parentheses).  ^c^ 0, HIIT partly or entirely replaced regular training; 1, HIIT was added to regular training. | | | | | | | | | | | | | | | |

| Table 7. Study and subject characteristics included in the meta-analysis of effects of HIIT on aerobic/anaerobic threshold for non-endurance trained athletes. | | | | | | | | | | | | | |
| --- | --- | --- | --- | --- | --- | --- | --- | --- | --- | --- | --- | --- | --- |
| **Study** | **Year** | **Type of athlete** | **Treatment abbreviation** | **Our treatment** | **Type of HIIT^a^** | **Sample size** | **Maleness** | **Phase of training^b^** | **Extra training^c^** | **Intervention duration (wk)** | **Threshold pre^d^** | **Effect (%)**  **Mean; 90% CL** |  |
| Breil et al. [19] | 2010 | Other | CT | Control |  | 8 | 0.75 | -1 | 0 | 2 | 53.6 | 0.7; -6.2 to 8.2 |  |
|  |  | Other | CT | Control |  | 8 | 0.75 | -1 | 0 | 2 | 84.5 | 1.2; -2.8 to 5.5 |  |
|  |  | Other | IT | HIIT | 1 | 13 | 0.69 | -1 | 0 | 2 | 57.8 | 2.1; -3.1 to 7.6 |  |
| Breil et al. [19] | 2010 | Other | IT | HIIT | 1 | 13 | 0.69 | -1 | 0 | 2 | 85.3 | 9.8; 6.6 to 13.2 |  |
| Clark et al. [2] | 2014 | Endurance | Control | Control |  | 9 | 1 | 0 | 0 | 1 | 84.6 | -3.4; -7.1 to 0.4 |  |
|  |  | Endurance | Short HIIT | HIIT | 5 | 9 | 1 | 0 | 0 | 1 | 78.9 | 3.8; 1.6 to 6.0 |  |
|  |  | Endurance | Long HIIT | HIIT | 4 | 10 | 1 | 0 | 0 | 1 | 87.3 | 2.7; -0.4 to 5.8 |  |
| Helgerud et al. [22] | 2001 | Other | CON | Control |  | 10 | 1 | 0 | 1 | 8 | 86.2 | -1.7; -7.6 to 4.5 |  |
|  |  | Other | HIIT | HIIT | 1 | 9 | 1 | 0 | 1 | 8 | 87.4 | 5.4; -1.3 to 12.6 |  |
| Rønnestad et al. [7] | 2019 | Endurance | Exp | HIIT | 3 | 9 | 1 | 0 | 1 | 0.9 | 69.5 | 3.9; 2.0 to 5.9 |  |
| Sandbakk et al. [9] | 2011 | Endurance | CON | Control |  | 8 | 0.33 | -1 | 0 | 8 | 80.9 | 0.0; -4.0 to 4.2 |  |
|  |  | Endurance | IG | HIIT | 1 | 7 | 0.33 | -1 | 0 | 8 | 77.2 | 9.8; 6.4 to 13.3 |  |
| Sandbakk et al. [10] | 2013 | Endurance | CON | Control |  | 7 | 0.57 | -1 | 1 | 8 | 79.0 | 1.2; -3.2 to 5.9 |  |
|  |  | Endurance | SIG | HIIT | 1 | 7 | 0.57 | -1 | 1 | 8 | 78.9 | 1.6; -2.8 to 6.3 |  |
|  |  | Endurance | LIG | HIIT | 1 | 7 | 0.57 | -1 | 1 | 8 | 77.0 | 9.8; 5.0 to 14.9 |  |
| Sheykhlouvand et al. [29] | 2018 | Other | Control | Control |  | 8 | 0 | -1 | 0 | 4 | 73.1 | 1.5; -2.5 to 5.8 |  |
|  |  | Other | SIT | HIIT | 5 | 8 | 0 | -1 | 0 | 4 | 77.7 | 7.5; 3.2 to 12.0 |  |
| Smith et al. [12] | 2003 | Endurance | CON | Control |  | 9 | (1) | . | 0 | 4 | 76.7 | 2.9; -0.9 to 6.9 |  |
|  |  | Endurance | 60%Tmax | HIIT | 1 | 9 | (1) | . | 0 | 4 | 73.5 | 15.7; 11.4 to 20.1 |  |
|  |  | Endurance | 70%Tmax | HIIT | 1 | 9 | (1) | . | 0 | 4 | 78.4 | 6.4; 2.5 to 10.5 |  |
| Stevens et al. [15] | 2015 | Endurance | EBTAlone | Control |  | 8 | 1 | -1 | 0 | 4 | 81.0 | 1.6; -5.4 to 9.1 |  |
|  |  | Endurance | EBTSIT | HIIT | 4 | 8 | 1 | -1 | 0 | 4 | 83.0 | -1.5; -8.3 to 5.7 |  |
| Stöggl et al. [16] | 2014 | Endurance | HVTcyc | Control |  | 3 | 1 | -0.5 | 0 | 9 | 59.6 | 4.0; -15.8 to 28.5 |  |
|  |  | Endurance | HVTrun | Control |  | 5 | 1 | -0.5 | 0 | 9 | 65.5 | -1.4; -15.6 to 15.1 |  |
|  |  | Endurance | HVTrun | Control |  | 5 | 1 | -0.5 | 0 | 9 | 82.9 | 1.8; -4.6 to 8.7 |  |
|  |  | Endurance | HVTcyc | Control |  | 3 | 1 | -0.5 | 0 | 9 | 80.0 | 0.1; -12.6 to 14.6 |  |
|  |  | Endurance | HIIT | HIIT | 1 | 9 | 1 | -0.5 | 0 | 9 | 51.0 | 11.3; 6.4 to 16.3 |  |
|  |  | Endurance | POLcyc | HIIT | 1 | 4 | 1 | -0.5 | 0 | 9 | 56.1 | 2.7; -9.3 to 16.3 |  |
|  |  | Endurance | POLrun | HIIT | 1 | 7 | 0.86 | -0.5 | 0 | 9 | 59.0 | 9.8; 3.2 to 16.9 |  |
|  |  | Endurance | HIIT | HIIT | 1 | 9 | 1 | -0.5 | 0 | 9 | 75.4 | 5.2; 2.4 to 8.1 |  |
|  |  | Endurance | POLrun | HIIT | 1 | 7 | 0.86 | -0.5 | 0 | 9 | 79.2 | 8.4; 4.6 to 12.4 |  |
|  |  | Endurance | POLcyc | HIIT | 1 | 4 | 1 | -0.5 | 0 | 9 | 79.3 | 6.6; 5.4 to 7.9 |  |
| Sylta et al. [17] | 2016 | Endurance | INC | HIIT | 1 | 23 | 1 | -1 | 0 | 12 | 77.3 | 5.1; 2.2 to 8.0 |  |
|  |  | Endurance | DEC | HIIT | 1 | 20 | 1 | -1 | 0 | 12 | 79.4 | 0.8; -1.7 to 3.4 |  |
|  |  | Endurance | MIX | HIIT | 1 | 20 | 1 | -1 | 0 | 12 | 80.7 | 3.3; 0.5 to 6.1 |  |
| CI, confidence intervals.  Excluded studies: none.  ^a^ Range of 1 (aerobic traditional long intervals) to 6 (anaerobic sprint intervals), as presented in Figure 1 in Stöggl et al. [38]  ^b^ 0, during the competition phase; 1, outside the competition phase; 0.5, mixture of competition and pre- or post-competition phase. Missing values were imputed to the mean of eligible study estimates (shown in parentheses).  ^c^ 0, HIIT partly or entirely replaced regular training; 1, HIIT was added to regular training.  ^d^ Threshold as a percentage of V̇O_2max_. | | | | | | | | | | | | | |

| Table 8. Study and subject characteristics included and excluded in the meta-analysis of effects of HIIT on **V̇O_2max_**. | | | | | | | | | | | | | |
| --- | --- | --- | --- | --- | --- | --- | --- | --- | --- | --- | --- | --- | --- |
| **Study** | **Year** | **Type of athlete** | **Authors’ treatment abbreviation** | **Our treatment** | **Type of HIIT^a^** | **Sample size** | **Maleness^b^** | **Phase of training^c^** | **Extra training^d^** | **Intervention duration (wk)** | **V̇O_2max_ pre**  **Mean ± SD** | **Effect (%)**  **Mean; 90% CL** |  |
| Breil et al. [19] | 2010 | Other | CT | Control |  | 8 | 0.75 | 1 | 0 | 2 | 52.9 ± 6.3 | 2.8; -0.4 to 6.2 |  |
|  |  | Other | IT | HIIT | 1 | 13 | 0.69 | 1 | 0 | 2 | 53.0 ± 4.6 | 6.0; 4.2 to 7.9 |  |
| Clark et al. [2] | 2014 | Endurance | Control | Control |  | 9 | 1 | 0 | 0 | 1 | 61.2 ± 10.0 | -0.8; -4.2 to 2.7 |  |
|  |  | Endurance | Short HIIT | HIIT | 5 | 9 | 1 | 0 | 0 | 1 | 65.4 ± 4.7 | 2.2; -0.7 to 5.2 |  |
|  |  | Endurance | Long HIIT | HIIT | 4 | 10 | 1 | 0 | 0 | 1 | 62.8 ± 5.2 | 3.7; 0.3 to 7.3 |  |
| Hanstock et al. [3] | 2020 | Endurance | Si1 | HIIT | 3 | 9 | 1 | 1 | 0 | 4 | 63.5 ± 5.6 | 3.6; 1.6 to 5.7 |  |
|  |  | Endurance | Si2 | HIIT | 3 | 9 | 1 | 1 | 0 | 4 | 64.5 ± 7.3 | 0.3; -0.6 to 1.2 |  |
| Helgerud et al. [22] | 2001 | Other | CON | Control |  | 10 | 1 | 0 | 1 | 8 | 58.4 ± 4.3 | 1.9; -0.9 to 4.7 |  |
|  |  | Other | HIIT | HIIT | 1 | 9 | 1 | 0 | 1 | 8 | 58.1 ± 4.5 | 10.7; 7.7 to 13.7 |  |
| Johansen et al. [4] | 2021 | Endurance | CON | Control |  | 7 | (1) | 1 | 0 | 6 | 71.6 ± 3.9 | -0.6; -3.5 to 2.5 |  |
|  |  | Endurance | HIIT | HIIT | 1 | 7 | (1) | 1 | 0 | 6 | 65.8 ± 10.9 | -3.8; -7.1 to -0.4 |  |
| Laursen et al. [5] | 2002 | Endurance | Control | Control |  | 11 | 1 | 1 | 0 | 4 | 65.2 ± 5.9 | 0.8; -0.9 to 2.6 |  |
|  |  | Endurance | G1 | HIIT | 1 | 8 | 1 | 1 | 1 | 4 | 66.5 ± 6.2 | 5.2; 2.9 to 7.6 |  |
| Laursen et al. [5] | 2002 | Endurance | G2 | HIIT | 1 | 9 | 1 | 1 | 1 | 4 | 63.7 ± 4.1 | 8.0; 6.0 to 10.0 |  |
|  |  | Endurance | G3 | HIIT | 4 | 10 | 1 | 1 | 1 | 4 | 62.6 ± 4.1 | 3.1; 1.4 to 4.8 |  |
| Liu et al. [25] | 2021 | Other | CON male | Control |  | 8 | 1 | 0 | 0 | 8 | 55.8 ± 8.0 | 3.4; 0.2 to 6.8 |  |
|  |  | Other | CON female | Control |  | 8 | 0 | 0 | 0 | 8 | 42.9 ± 1.6 | 0.9; -2.2 to 4.2 |  |
|  |  | Other | SIT male | HIIT | 4 | 8 | 1 | 0 | 0 | 8 | 56.8 ± 7.0 | 12.0; 8.8 to 15.3 |  |
|  |  | Other | SIT female | HIIT | 4 | 8 | 0 | 0 | 0 | 8 | 42.5 ± 2.9 | 8.7; 5.6 to 11.9 |  |
| Menz et al. [6] | 2015 | Endurance | HIIT | HIIT | 1 | 19 | 0.74 | 1 | 0 | 3 | 63.6 ± 7.5 | 3.5; 1.3 to 5.6 |  |
| Rønnestad et al. [7] | 2019 | Endurance | Exp | HIIT | 3 | 9 | 1 | 0 | 1 | 0.9 | 76.8 ± 6.9 | 3.8; 2.1 to 5.5 |  |
| Salazar-Martínez et al. [8] | 2018 | Endurance | Con | Control |  | 8 | 1 | 1 | 0 | 3 | 67.0 ± 6.5 | -0.3; -3.6 to 3.1 |  |
|  |  | Endurance | HIIT | HIIT | 1 | 8 | 1 | 1 | 0 | 3 | 68.4 ± 2.7 | 2.0; -0.9 to 5.1 |  |
| Sandbakk et al. [9] | 2011 | Endurance | CON | Control |  | 8 | 0.33 | 1 | 0 | 8 | 69.3 ± 7.2 | 1.4; -2.2 to 5.2 |  |
|  |  | Endurance | IG | HIIT | 1 | 7 | 0.33 | 1 | 0 | 8 | 67.5 ± 6.5 | 4.0; 2.5 to 5.6 |  |
| Sandbakk et al. [10] | 2013 | Endurance | CON | Control |  | 7 | 0.57 | 1 | 1 | 8 | 68.0 ± 7.0 | 0.0; -2.7 to 2.8 |  |
|  |  | Endurance | SIG | HIIT | 1 | 7 | 0.57 | 1 | 1 | 8 | 67.0 ± 8.0 | 3.0; 0.6 to 5.4 |  |
|  |  | Endurance | LIG | HIIT | 1 | 7 | 0.57 | 1 | 1 | 8 | 67.0 ± 6.0 | 4.5; 3.3 to 5.7 |  |
| Sheykhlouvand et al. [28] | 2016 | Other | Gcon | Control |  | 7 | (1) | 1 | 0 | 3 | 37.1 ± 5.7 | 2.2; -1.8 to 6.3 |  |
|  |  | Other | G1 | HIIT | 3 | 7 | (1) | 1 | 0 | 3 | 39.2 ± 4.1 | 6.6; 3.0 to 10.3 |  |
|  |  | Other | G2 | HIIT | 3 | 7 | (1) | 1 | 0 | 3 | 37.8 ± 4.6 | 7.7; 2.8 to 12.8 |  |
| Sheykhlouvand et al. [29] | 2018 | Other | Control | Control |  | 8 | 0 | 1 | 0 | 4 | 39.2 ± 4.1 | 1.5; 0.0 to 3.1 |  |
| Sheykhlouvand et al. [29] | 2018 | Other | SIT | HIIT | 5 | 8 | 0 | 1 | 0 | 4 | 37.1 ± 3.5 | 7.5; 3.3 to 11.9 |  |
| Skovereng et al. [11] | 2018 | Endurance | HIIT | HIIT | 1 | 60 | 1 | 1 | 1 | 12 | 61.0 ± 6.0 | 6.6; 5.9 to 7.2 |  |
| Smith et al. [12] | 2003 | Endurance | CON | Control |  | 9 | (1) | (0.68) | 0 | 4 | 63.6 ± 6.0 | 0.6; -2.3 to 3.6 |  |
|  |  | Endurance | 60%Tmax | HIIT | 1 | 9 | (1) | (0.68) | 0 | 4 | 60.5 ± 5.7 | 6.0; 3.1 to 8.8 |  |
|  |  | Endurance | 70%Tmax | HIIT | 1 | 9 | (1) | (0.68) | 0 | 4 | 60.1 ± 1.8 | 4.2; 1.4 to 7.0 |  |
| Stenqvist et al. [13] | 2020 | Endurance | HIIT | HIIT | 2 | 22 | (1) | 1 | 0 | 4 | 63.5 ± 6.6 | 2.4; 1.1 to 3.7 |  |
| Stevens et al. [15] | 2015 | Endurance | EBTAlone | Control |  | 8 | (1) | 1 | 0 | 4 | 63.3 ± 6.2 | -1.7; -4.8 to 1.5 |  |
|  |  | Endurance | EBTSIT | HIIT | 4 | 8 | (1) | 1 | 0 | 4 | 61.4 ± 7.4 | 0.0; -2.9 to 3.0 |  |
| Stöggl et al. [16] | 2014 | Endurance | HVTcyc | Control |  | 3 | 1 | 0.5 | 0 | 9 | 55.8 ± 4.2 | 3.4; -0.4 to 7.4 |  |
|  |  | Endurance | HVTrun | Control |  | 5 | 1 | 0.5 | 0 | 9 | 63.4 ± 10.9 | 2.0; -3.4 to 7.7 |  |
|  |  | Endurance | HIIT | HIIT | 1 | 9 | 1 | 0.5 | 0 | 9 | 63.7 ± 7.1 | 4.5; 1.0 to 8.0 |  |
|  |  | Endurance | POLcyc | HIIT | 1 | 7 | 1 | 0.5 | 0 | 9 | 61.5 ± 8.3 | 11.6; 5.5 to 18.1 |  |
|  |  | Endurance | POLrun | HIIT | 1 | 4 | 0.75 | 0.5 | 0 | 9 | 59.1 ± 9.3 | 10.3; 6.4 to 14.4 |  |
| Sylta et al. [17] | 2016 | Endurance | INC | HIIT | 1 | 23 | 1 | 1 | 0 | 12 | 61.8 ± 5.4 | 7.8; 5.9 to 9.6 |  |
|  |  | Endurance | DEC | HIIT | 1 | 20 | 1 | 1 | 0 | 12 | 60.6 ± 4.1 | 5.8; 3.9 to 7.6 |  |
|  |  | Endurance | MIX | HIIT | 1 | 20 | 1 | 1 | 0 | 12 | 61.6 ± 3.8 | 4.9; 3.1 to 6.7 |  |
| Wells et al. [34] | 2014 | Other | CON | Control |  | 8 | 1 | 0 | 0 | 6 | 57.1 ± 3.6 | 0.9; -3.7 to 5.6 |  |
|  |  | Other | TRA | HIIT | 4 | 8 | 1 | 0 | 0 | 6 | 57.6 ± 5.4 | 2.3; -4.5 to 9.5 |  |
| Yang et al. [35] | 2017 | Other | HIIT | HIIT | 2 | 7 | 1 | 1 | 0 | 4 | 45.9 ± 7.2 | 6.5; 3.1 to 10.1 |  |
| **Excluded studies** | | | | | | | | | | | | | |
| Sheykhlouvand et al. [30] | 2018 | Other | CET | Control |  | 7 | 1 | 1 | 0 | 3 | 37.1 ± 5,7 | 2.2; -1.8 to 6.3 |  |
|  |  | Other | VVHIIT | HIIT | 3 | 7 | 1 | 1 | 0 | 3 | 39.2 ± 4.1 | 6.6.; 3.0 to 10.3 |  |
|  |  | Other | VIHIIT | HIIT | 3 | 7 | 1 | 1 | 0 | 3 | 37.8 ± 4.6 | 7.7; 2.8 to 12.8 |  |
| CI, confidence intervals.  Excluded studies: equal V̇O_2max_ values as in Sheykhlouvand, Khallili [28]  ^a^ Range of 1 (aerobic traditional long intervals) to 6 (anaerobic sprint intervals), as presented in Figure 1 in Stöggl et al. [38]  ^b^ Proportion of males; missing values were imputed to 1 (shown in parentheses).  ^c^ 0, during the competition phase; 1, outside the competition phase; 0.5, mixture of competition and pre- or post-competition phase. Missing values were imputed to the mean of eligible study estimates (shown in parentheses).  ^d^ 0, HIIT partly or entirely replaced regular training; 1, HIIT was added to regular training. | | | | | | | | | | | | | |

| Table 9. Study and subject characteristics included in the meta-analysis of effects of HIIT on **exercise economy** for endurance-trained athletes. | | | | | | | | | | | |  |
| --- | --- | --- | --- | --- | --- | --- | --- | --- | --- | --- | --- | --- |
| **Study** | **Year** | **Type of athlete** | **Treatment abbreviation** | **Our treatment** | **Type of HIIT^a^** | **Sample size** | **Maleness** | **Phase of training^b^** | **Intervention duration (wk)** | **Effect (%)**  **Mean; 90% CL** |  |  |
| Clark et al. [2] | 2014 | Endurance | Control | Control |  | 9 | 1 | 0 | 1 | 2.2; -0.4 to 4.9 |  |  |
|  |  | Other | Short HIIT | HIIT | 5 | 9 | 1 | 0 | 1 | 3.8; 1.1 to 6.5 |  |  |
|  |  | Endurance | Long HIIT | HIIT | 4 | 10 | 1 | 0 | 1 | 4.7; 2.5 to 7.0 |  |  |
| Helgerud et al. [22] | 2001 | Other | CON | Control |  | 10 | 1 | 0 | 8 | 1.4; -3.3 to 6.3 |  |  |
|  |  | Other | HIIT | HIIT | 1 | 9 | 1 | 0 | 8 | 7.1; 1.9 to 12.7 |  |  |
| Rønnestad et al. [7] | 2019 | Endurance | Exp | HIIT | 3 | 9 | 1 | 0 | 0.86 | 0.4; -4.9 to 6.0 |  |  |
| Skovereng et al. [11] | 2018 | Endurance | HIIT | HIIT | 1 | 60 | 1 | -1 | 12 | -0.4; -1.4 to 0.5 |  |  |
| Stöggl et al. [16] | 2014 | Endurance | HVTcyc | Control |  | 3 | 1 | -0.5 | 9 | -0.1; -16.0 to 18.7 |  |  |
|  |  | Endurance | HVTrun | Control |  | 5 | 1 | -0.5 | 9 | -0.1; -9.5 to 10.1 |  |  |
|  |  | Endurance | HIIT | HIIT | 1 | 9 | 1 | -0.5 | 9 | -3.1; -10.1 to 4.5 |  |  |
|  |  | Endurance | POLcyc | HIIT | 1 | 6 | 1 | -0.5 | 9 | -3.9; -11.5 to 4.3 |  |  |
| Sylta et al. [17] | 2016 | Endurance | INC | HIIT | 1 | 23 | 1 | -1 | 12 | -0.5; -2.4 to 1.5 |  |  |
|  |  | Endurance | DEC | HIIT | 1 | 20 | 1 | -1 | 12 | -0.4; -2.1 to 1.3 |  |  |
|  |  | Endurance | MIX | HIIT | 1 | 20 | 1 | -1 | 12 | -0.2; -1.9 to 1.5 |  |  |
| Yang et al. [35] | 2017 | Other | HIIT | HIIT | 1.5 | 7 | 1 | -1 | 4 | 3.1; -2.9 to 9.5 |  |  |
| **Excluded study estimate** | | | | | | | | | | | | |
| Stöggl et al. [16] | 2014 |  | POLrun | HIIT | 1 | 6 | 0.83 | -0.5 | 9 | -3.9; -11.5 to 4.3 |  |  |
| CI, confidence intervals.  Excluded studies: none.  ^a^ Range of 1 (aerobic traditional long intervals) to 6 (anaerobic sprint intervals), as presented in Figure 1 in Stöggl et al. [38]  ^b^ 0, during the competition phase; 1, outside the competition phase; 0.5, mixture of competition and pre- or post-competition phase. Missing values were imputed to the mean of eligible study estimates (shown in parentheses). | | | | | | | | | | | |  |

| Table 10: Summary of Tables 4 to 10 in the original manuscript, including predicted effects, moderator effects, and heterogeneity SDs in the meta-analyses of each performce measure. Error of measurement and individual-responses SDs are also included. The data are effect qualitative magnitude, probability of the magnitude, and the observed quantitative magnitude. | | | | | | | | | | | | | | | | | | | | |
| --- | --- | --- | --- | --- | --- | --- | --- | --- | --- | --- | --- | --- | --- | --- | --- | --- | --- | --- | --- | --- |
|  | | **Sprint speed/ power** | | **Repeated sprint ability** | | **Time-trial speed/ power** | | **Peak speed/ power** | | **Aerobic/ anaerobic threshold** | | | **V̇O_2max_** | | | | **Exercise economy** | | | |
| ***Predicted effects*** | | **^a^** | | **^b^** | | **^c^** | | **^d^** | | **^e^** | | | **^f^** | | | | **^g^** | | | |
| **Endurance athletes** | |  | |  | |  | |  | |  | | |  | | | |  | | | |
|  | Males HIIT |  |  |  |  | ↑**** | 4.8 | **↑****** | 3.4 | **↑****** | 5.5 | | **↑****** | | 7.9 | |  | | 1.1 | |
|  | Males control |  |  |  |  | ↓*↔^0^ | -0.5 |  | 0.3 |  | 0.7 | | ↔^0^ | | 0.1 | |  | | 1.5 | |
|  | HIIT – control |  |  |  |  | ↑*** | 5.3 | **↑***** | 3.1 | ↑*** | 4.8 | | **↑****** | | 7.7 | |  | | -0.3 | |
|  | Females HIIT |  |  |  |  |  |  |  |  | ↑*** | 12.3 | | **↑****** | | 7.1 | |  | |  | |
|  | Females control |  |  |  |  |  |  |  |  |  | -0.2 | |  | | -0.3 | |  | |  | |
|  | HIIT – control |  |  |  |  |  |  |  |  | ↑*** | 12.6 | | ↑**** | | 7.4 | |  | |  | |
| **Other athletes** | |  | |  | |  | |  | |  | | |  | | | |  | |  | |
|  | Males HIIT | ↑*** | 4.2 | ↑*** | 3.2 |  |  | ↔0 | 0.0 | ↑** | 6.1 | | ↑**** | | 11.1 | |  | | 1.1 | |
|  | Males control | ↓** | -2.3 | ↓*↔0 | -0.7 |  |  | ↓*↔0 | -1.3 |  | 1.0 | | ↑** | | 2.3 | |  | | 1.5 | |
|  | HIIT – control | ↑*** | 6.7 | ↑** | 3.9 |  |  |  | 1.3 |  | 5.1 | | ↑**** | | 8.6 | |  | | -0.3 | |
|  | Females HIIT |  |  |  | -1.1 |  |  |  |  | ↑** | 7.6 | | ↑**** | | 12.5 | |  | |  | |
|  | Females control |  |  | ↓* | -2.2 |  |  |  |  |  | 1.4 | |  | | 0.9 | |  | |  | |
|  | HIIT – control |  |  |  | 1.1 |  |  |  |  |  | 6.2 | | ↑**** | | 11.5 | |  | |  | |
|  | Males YoYo HIIT |  |  |  |  |  |  | ↑** | 1.7 |  |  | |  | |  | |  | |  | |
|  | Males YoYo control |  |  |  |  |  |  |  | 0.5 |  |  | |  | |  | |  | |  | |
|  | HIIT – control |  |  |  |  |  |  |  | 1.2 |  |  | |  | |  | |  | |  | |
|  | | | | | | | | | | | | | | | | |  | |  | |
|  | | | | | | | | | | | | | | | | |  | |  | |
| ***Moderator effects*** | |  | |  | |  | |  | |  | | |  | | | |  | |  | |
| **Male – female athletes for HIIT – control** | | | | | | | | | | | | | | | | |  | |  | |
|  | Endurance athletes |  |  |  |  |  |  |  |  |  | -6.9 | |  | | -0.3 | |  | |  | |
|  | Other athletes |  |  |  | 2.8 |  |  |  |  |  | -1.0 | |  | | -2.6 | |  | |  | |
| **Endurance – other athletes for HIIT – control** | | | | | | | | | | | | | | | | |  | |  | |
|  | Males |  |  |  |  |  |  |  | 1.7 |  | 0.3 | |  | | -0.8 | |  | |  | |
|  | Females |  |  |  |  |  |  |  |  |  | 6.0 | |  | | -3.7 | |  | |  | |
| **Yoyo – Incr. for HIIT – control** | | | | | | | | | | | | | | | | |  | |  | |
|  | Males |  |  |  |  |  |  |  | -0.2 |  |  | |  | |  | |  | |  | |
| **Type of HIIT^h^** | |  |  |  |  |  |  |  |  |  |  | |  | |  | |  | |  | |
|  | Type 4.0 vs 1.0 HIIT |  | -0.4 |  | -1.0 |  | 0.4 | ↔^00^ | -0.3 |  | -2.7 | | **↓***** | | -2.6 | |  | |  | |
| **Intervention duration** | | +5^g^ wk | | +4 wk | | +6 wk | | +8 wk | | +7 wk | | | +5 wk | | | |  | |  | |
|  | HIIT | ↔^0^↑* | 0.7 | ↑** | 2.7 |  | 0.5 | ↔^0^↑* | 1.0 | ↓** | -1.3 | | ↔^0^**↑*** | | 1.2 | |  | |  | |
|  | Control |  | -0.9 |  | 2.4 |  | 5.2 |  | 2.1 |  | 2.1 | | ↑* | | 1.7 | |  | |  | |
|  | HIIT – control | ↔^0^↑* | 1.6 |  | 0.3 |  | 4.5 |  | -1.1 | ↓** | -3.3 | |  | | -0.6 | |  | |  | |
| **Training phase** | |  | |  | |  |  |  |  |  |  | |  | | | |  | |  | |
|  | On-season HIIT | ↑******* | 2.6 |  | 1.2 |  |  |  |  |  |  | | ↑******* | | 3.6 | |  | |  | |
|  | On-season cont | ↑*↔^0^ | 1.6 |  | -1.1 |  |  |  |  |  |  | |  | | -0.6 | |  | |  | |
|  | On-season HIIT – control |  | 1.0 |  | 2.2 |  |  |  |  |  |  | | ↑******* | | 4.3 | |  | |  | |
| **Training implementation** | |  |  |  |  |  |  |  |  |  |  | |  | |  | |  | |  | |
|  | HIIT as extra training | ↔^0^↑* | 1.3 |  | 0.4 |  | 1.8 | ↔^0^↑* | 1.2 |  | -1.6 | | ↔^0^↑* | | 1.1 | |  | |  | |
|  | Extra control training |  |  |  |  |  |  |  |  |  | -0.8 | |  | | -1.2 | |  | |  | |
|  | HIIT – control |  |  |  |  |  |  |  |  |  | -0.8 | | **↑**** | | 2.3 | |  | |  | |
| ***Pre-test moderators*** | |  | |  |  |  | |  | |  |  |  | |  | |  | |  | |  |
| **Test duration:** | | 10 vs 2.5 s | |  |  | +40 min | | +8 min | |  |  |  | |  | |  | |  | |  |
|  | HIIT | ↑*** | 2.9 |  |  | **↑**** | 2.7 | ↔^0^↑* | 0.4 |  |  | |  | |  | |  | |  | |
|  | Control |  | -2.5 |  |  | ↓** | -2.1 |  | -0.8 |  |  | |  | |  | |  | |  | |
|  | HIIT – control | ↑*** | 5.5 |  |  | **↑***** | 4.9 |  | 1.2 |  |  | |  | |  | |  | |  | |
| **Other measures:** | |  |  | Decr.^g^ +3% | |  |  |  |  | Threshold +10% | | | V̇O_2max_ +20% | | | | Intensity +20% | | | |
|  | HIIT |  |  | ↑*** | 5.3 |  |  |  |  | ↓*↔^0^ | -0.8 | | **↓**** | | -1.8 | |  | | 0.3 | |
|  | Control |  |  |  | 1.2 |  |  |  |  |  | -0.7 | |  | | -0.4 | |  | | 0.0 | |
|  | HIIT – control |  |  |  | 4.0 |  |  |  |  |  | 0.0 | | ↓* ↔^0^ | | -1.4 | |  | | 0.2 | |
|  |  |  |  |  |  |  |  |  |  |  |  | |  | |  | |  | |  | |
|  | |  |  |  |  |  |  |  |  |  |  | |  | |  | |  | |  | |
| ***Heterogeneity SD*** | |  |  |  |  |  |  |  |  |  |  | |  | |  | |  | |  | |
|  | HIIT | ↑** | 1.2 |  | 1.2 | ↑** | 1.3 |  | 1.3 |  | 2.3 | |  | | 1.1 | |  | | 2.2 | |
|  | Control |  | 0.6 |  | 2.3 |  | 0.7 |  | 1.8 |  | 1.0 | |  | | 0.4 | |  | |  |  |
|  |  |  |  |  |  |  |  |  |  |  |  | |  | |  | |  | |  | |
|  | |  |  |  |  |  |  |  |  |  |  | |  | |  | |  | |  | |
| ***Error of measurement SD^i^*** | |  |  |  |  |  |  |  |  |  |  | |  | |  | |  | |  | |
|  | HIIT |  | 2.2 |  | 1.4 |  | 3.2 |  | 2.9 |  | 3.5 | |  | | 3.1 | |  | | 6.0 | |
|  | Control |  | 1.9 |  | 1.3 |  | 1.9 |  | 1.9 |  | 4.4 | |  | | 3.4 | |  | | 3.6 | |
| ***Individual responses SD^j^*** | |  | 2.2 |  | 0.7 |  | 3.6 |  | 3.1 |  | -3.8 | |  | | -2.0 | |  | | 6.8 | |
| V̇O_2max_, maximum oxygen uptake; Decr., repeated-sprint ability decrement.  Sign and probability are shown for effects with adequate precision at the 90% or 99% level. ↑↓ Indicate substantial positive and negative effects, respectively; ↔ indicates trivial effects. Probabilities of substantial effects: *, possibly; **, likely; ***, very likely; ****, most likely. Probabilities of trivial effects: ^0^, possibly; ^00^, likely; ^000^, very likely; ^0000^, most likely.  Predicted effects (a-g) and other explanations (h-j):  ^a^ 10-s test; HIIT as replacement training, on-season; six weeks; aerobic rank 3  ^b^ decrement 5%; HIIT as replacement training, on-season; five weeks; aerobic rank 4  ^c^ 45-m test, HIIT as replacement training, on-season; five weeks; aerobic rank 2  ^d^ 8-min incremental test; HIIT as replacement training, on-season; five weeks; aerobic rank 3  ^e^ pre-test intensity of 80% of V̇O_2max_; HIIT as replacement training, off-season; six weeks; aerobic rank 1  ^f^ pre-test V̇O2max, 63 ml∙kg-1∙min-1 for female and male endurance athletes, 41 and 51 ml∙kg-1∙min-1 for female and male other athletes; HIIT as replacement training, on-season; five weeks; aerobic rank 1  ^g^ exercise-test intensity, 70% of V̇O_2max_  ^h^ Range of 1.0 (aerobic HIIT with traditional long intervals) to 6.0 (SIT: anaerobic sprint intervals), as presented in Figure 1 in Wiesinger et al. [36]  ^i^ Averaged across studies, where they could be estimated.  ^j^ Estimated as √(2e_H_^2^ – 2e_C_^2^), where e_H_ and e_C_ are the errors of measurement in HIIT and control groups, respectively. | | | | | | | | | | | | | | | | | | | | |

**References**

1. Galna B, Peters A, Murphy AT, Morris ME. Obstacle crossing deficits in older adults: a systematic review. Gait Posture. 2009 Oct;30(3):270-5.

2. Clark B, Costa VP, O'Brien BJ, Guglielmo LG, Paton CD. Effects of a seven day overload-period of high-intensity training on performance and physiology of competitive cyclists. PLoS One. 2014;9(12):e115308.

3. Hanstock HG, Govus AD, Stenqvist TB, Melin AK, Sylta Ø, Torstveit MK. Influence of immune and nutritional biomarkers on illness risk during interval training. Int J Sports Physiol Perform. 2020 2020;15(1):60-7.

4. Johansen JM, Eriksen S, Sunde A, Slettemeas OB, Helgerud J, Storen O. Improving utilization of maximal oxygen uptake and work economy in recreational cross-country skiers with high-intensity double-poling Intervals. Int J Sports Physiol Perform. 2021 Jan 1;16(1):37-44.

5. Laursen PB, Shing CM, Peake JM, Coombes JS, Jenkins DG. Interval training program optimization in highly trained endurance cyclists. Med Sci Sports Exerc. 2002;34(11):1801-7.

6. Menz V, Strobl J, Faulhaber M, Gatterer H, Burtscher M. Effect of 3-week high-intensity interval training on VO2max, total haemoglobin mass, plasma and blood volume in well-trained athletes. Eur J Appl Physiol. 2015 Nov;115(11):2349-56.

7. Rønnestad BR, Vikmoen O. A 11-day compressed overload and taper induces larger physiological improvements than a normal taper in elite cyclists. Scand J Med Sci Sports. 2019 Dec;29(12):1856-65.

8. Salazar-Martinez E, Santalla A, Orellana JN, Strobl J, Burtscher M, Menz V. Influence of high-intensity interval training on ventilatory efficiency in trained athletes. Respir Physiol Neurobiol. 2018 Apr;250:19-23.

9. Sandbakk Ø, Welde B, Holmberg HC. Endurance training and sprint performance in elite junior cross-country skiers. J Strength Cond Res. 2011;25(5):1299-305.

10. Sandbakk Ø, Sandbakk SB, Ettema G, Welde B. Effects of intensity and duration in aerobic high-intensity interval training in highly trained junior cross-country skiers. J Strength Cond Res. 2013 Jul;27(7):1974-80.

11. Skovereng K, Sylta Ø, Tønnessen E, Hammarström D, Danielsen J, Seiler S, et al. Effects of initial performance, gross efficiency and V˙O2peak characteristics on subsequent adaptations to endurance training in competitive cyclists. Front Physiol. 2018 2018;9:713.

12. Smith TP, Coombes JS, Geraghty DP. Optimising high-intensity treadmill training using the running speed at maximal O(2) uptake and the time for which this can be maintained. Eur J Appl Physiol. 2003 May;89(3-4):337-43.

13. Stenqvist TB, Torstveit MK, Faber J, Melin AK. Impact of a 4-week intensified endurance training intervention on markers of relative energy deficiency in sport (RED-S) and performance among well-trained male cyclists. Front Endocrinol (Lausanne). 2020;11:512365.

14. Stepto NK, Hawley JA, Dennis SC, Hopkins WG. Effects of different interval-training programs on cycling time-trial performance. Med Sci Sports Exerc. 1999 1999;31(5):736-41.

15. Stevens AWJ, Olver TT, Lemon PWR. Incorporating sprint training with endurance training improves anaerobic capacity and 2,000-m erg performance in trained oarsmen. J Strength Cond Res. 2015;29(1):22-8.

16. Stöggl T, Sperlich B. Polarized training has greater impact on key endurance variables than threshold, high intensity, or high volume training. Front Physiol. 2014;5:33.

17. Sylta O, Tonnessen E, Hammarstrom D, Danielsen J, Skovereng K, Ravn T, et al. The effect of different high-intensity periodization models on endurance adaptations. Med Sci Sports Exerc. 2016 2016;48(11):2165-74.

18. Akdoğan E, Yılmaz İ, Köklü Y, Alemdaroğlu U, Cerrah AO. The effect of isolated or combined small-sided games and speed endurance training on physical performance parameters in young soccer players. Kinesiology. 2021;53(1):78-85.

19. Breil FA, Weber SN, Koller S, Hoppeler H, Vogt M. Block training periodization in alpine skiing: effects of 11-day HIT on VO2max and performance. Eur J Appl Physiol. 2010 Aug;109(6):1077-86.

20. Chtara M, Rouissi M, Haddad M, Chtara H, Chaalali A, Owen A, et al. Specific physical trainability in elite young soccer players: efficiency over 6 weeks' in-season training. Biol Sport. 2017 Jun;34(2):137-48.

21. Dupont G, Akakpo K, Berthoin S. The effect of in-season, high-intensity interval training in soccer players. J Strength Cond Res. 2004;18:584-9.

22. Helgerud J, Engen LC, Wisløff U, Hoff J. Aerobic endurance training improves soccer performance. Med Sci Sports Exerc. 2001 2001;33(11):1925-31.

23. Hermassi S, Ingebrigtsen J, Schwesig R, Fieseler G, Delank KS, Chamari K, et al. Effects of in-season short-term aerobic and high-intensity interval training program on repeated sprint ability and jump performance in handball players. J Sports Med Phys Fitness. 2018 2018;58(1):50-6.

24. Iaia FM, Fiorenza M, Perri E, Alberti G, Millet GP, Bangsbo J. The effect of two speed endurance training regimes on performance of soccer players. PLoS ONE. 2015 2015;10(9):e0138096.

25. Liu H, Leng B, Li Q, Liu Y, Bao D, Cui Y. The effect of eight-week sprint interval training on aerobic performance of elite badminton players. Int J Environ Res Public Health. 2021 Jan 13;18(2):638.

26. Purkhus E, Krustrup P, Mohr M. High-intensity training improves exercise performance in elite women volleyball players during a competitive season. J Strength Cond Res. 2016 Nov;30(11):3066-72.

27. Selmi W, Rebai H, Chtara M, Naceur A, Sahli S. Self-confidence and affect responses to short-term sprint interval training. Physiol Behav. 2018 May 1;188:42-7.

28. Sheykhlouvand M, Khallili E, Gharaat M. Hormonal and physiological adaptations to high-intensity interval training in professional male canoe polo athletes. J Strength Cond Res. 2016;30(3):859-66.

29. Sheykhlouvand M, Khalili E, Gharaat M, Arazi H, Khalafi M, Tarverdizadeh B. Practical model of low-volume paddling-based sprint interval training improves aerobic and anaerobic performances in professional female canoe polo athletes. J Strength Cond Res. 2018 2018;32(8):2375-82.

30. Sheykhlouvand M, Gharaat M, Khalili M, Agha-Alinejad H, Rahmaninia F, Arazi H. Low-volume high-intensity interval versus continuous endurance training: effects on hematological and cardiorespiratory system adaptations in professional canoe polo athletes. J Strength Cond Res. 2018;37(7):1852-60.

31. Soares-Caldeira LF, de Souza EA, de Freitas VH, de Moraes SM, Leicht AS, Nakamura FY. Effects of additional repeated sprint training during preseason on performance, heart rate variability, and stress symptoms in futsal players: a randomized controlled trial. J Strength Cond Res. 2014 Oct;28(10):2815-26.

32. Thomassen M, Christensen PM, Gunnarsson TP, Nybo L, Bangsbo J. Effect of 2-wk intensified training and inactivity on muscle Na+-K+ pump expression, phospholemman (FXYD1) phosphorylation, and performance in soccer players. J Appl Physiol (1985). 2010 Apr;108(4):898-905.

33. Venturelli M, Bishop D, Pettene L. Sprint training in preadolescent soccer players. Int J Sports Physiol Perform. 2008 2008;3(4):558-62.

34. Wells C, Edwards A, Fysh M, Drust B. Effects of high-intensity running training on soccer-specific fitness in professional male players. Appl Physiol Nutr Metab. 2014 Jul;39(7):763-9.

35. Yang MT, Lee MM, Hsu SC, Chan KH. Effects of high-intensity interval training on canoeing performance. Eur J Sport Sci. 2017 Aug;17(7):814-20.

36. Wiesinger H-P, Stöggl T, Haller N, Blumkaitis J, Strepp T, Kilzer F, et al. Meta-analyses of the effects of high-intensity interval training in elite athletes – part I: mean effects on various performance measures. Front Physiol. 2024;15.

37. Sarkar S, Debnath M, Das M, Bandyopadhyay A, Dey SK, Datta G. Effect of high intensity interval training on antioxidant status, inflammatory response and muscle damage indices in endurance team male players. Apunts Sports Medicine. 2021;56(210):100352.

38. Stöggl TL, Strepp T, Wiesinger H-P, Haller N. A training goal-oriented categorization model of high-intensity interval training. Front. Physiol. 2024; 15:1414307.
